# Divergence in function and expression of the NOD26-like intrinsic proteins in plants

**DOI:** 10.1186/1471-2164-10-313

**Published:** 2009-07-15

**Authors:** Qingpo Liu, Huasen Wang, Zhonghua Zhang, Jiasheng Wu, Ying Feng, Zhujun Zhu

**Affiliations:** 1 School of Agriculture and Food Science, Zhejiang Forestry University, Lin'an, Hangzhou 311300, PR China; 2 Institute of Vegetables and Flowers, Chinese Academy of Agricultural Sciences, Beijing 100081, PR China; 3 School of Forestry and Biotechnology, Zhejiang Forestry University, Lin'an, Hangzhou 311300, PR China; 4 College of Environmental and Resources Science, Zhejiang University, Hangzhou 310029, PR China

## Abstract

**Background:**

NOD26-like intrinsic proteins (NIPs) that belong to the aquaporin superfamily are plant-specific and exhibit a similar three-dimensional structure. Experimental evidences however revealed that functional divergence should have extensively occurred among NIP genes. It is therefore intriguing to further investigate the evolutionary mechanisms being responsible for the functional diversification of the NIP genes. To better understand this process, a comprehensive analysis including the phylogenetic, positive selection, functional divergence, and transcriptional analysis was carried out.

**Results:**

The origination of NIPs could be dated back to the primitive land plants, and their diversification would be no younger than the emergence time of the moss *P. patens*. The rapid proliferation of NIPs in plants may be primarily attributed to the segmental chromosome duplication produced by polyploidy and tandem duplications. The maximum likelihood analysis revealed that *NIPs *should have experienced strong selective pressure for adaptive evolution after gene duplication and/or speciation, prompting the formation of distinct *NIP *groups. Functional divergence analysis at the amino acid level has provided strong statistical evidence for shifted evolutionary rate and/or radical change of the physiochemical properties of amino acids after gene duplication, and DIVERGE2 has identified the critical amino acid sites that are thought to be responsible for the divergence for further investigation. The expression of plant NIPs displays a distinct tissue-, cell-type-, and developmental specific pattern, and their responses to various stress treatments are quite different also. The differences in organization of *cis*-acting regulatory elements in the promoter regions may partially explain their distinction in expression.

**Conclusion:**

A number of analyses both at the DNA and amino acid sequence levels have provided strong evidences that plant NIPs have suffered a high divergence in function and expression during evolution, which is primarily attributed to the strong positive selection or a rapid change of evolutionary rate and/or physiochemical properties of some critical amino acid sites.

## Background

The aquaporins are a family of small pore-forming integral membrane proteins, which contain six membrane-spanning α-helices, with the N- and C-termini facing the cytosol [1]. On the basis of the first sequenced member – the *M*ajor *I*ntrinsic *P*rotein of bovine lens cells [2], this family is also referred to as the MIP superfamily. The MIP proteins are found to be present in all kingdoms from archaea to plants and animals [1]. However, MIPs constitute a larger and more diverse family in plants than in animals. There are 35 and 39 MIP genes in the genomes of the model plants *Arabidopsis *[3] and rice [4] respectively. By contrast, mammals only possess 13 distinct MIPs [5]. According to sequence similarities, plant aquaporins are clearly classified into five major subfamilies: plasma-membrane intrinsic proteins (PIPs), tonoplast intrinsic proteins (TIPs), NOD26-like intrinsic proteins (NIPs), small basic intrinsic proteins (SIPs), and the GlpF-like intrinsic proteins (GIPs). The divergence of plant aquaporins into five subfamilies had been already established as early as the time of emergence of primitive terrestrial plants [6,7]. The last subfamily of MIP genes has so far only been identified in two mosses [8]. Within each of the other subfamilies, MIP genes can be further subdivided into more than two groups [9] that may correspond to their localization and transport selectivity [10].

There are strong evidences that aquaporins are central components in plant water relations [10,11]. The significances of plant aquaporins functioning in mediating water and/or other small solutes across biomembrane and response to external environmental stresses have been widely reported [12-14]. It is likely that the particular abundance of MIP genes in plants may be attributed to the higher degree of compartmentalization of plant cells and their greater necessity for fine-tuned water control [3]. Alternatively, the extensive proliferation of aquaporin isoforms may offer an adaptive advantage for plants to grow in different environmental conditions, possibly as a result of divergent transport selectivities or regulatory mechanisms [11].

NIPs that were defined as NOD26-like intrinsic proteins on the basis of the archetype nodulin26 protein identified firstly in soybean [15], are unique to plants. These proteins are presumed to be involved in exchange of metabolites between the host and the symbiont [1]. Nevertheless, NIPs are widely distributed in both leguminous and nonleguminous plants, indicating that plant NIP function is not limited to the role that they play in nodule symbiosis [16]. Of plant aquaporins, only proteins belonging to this subfamily have glycerol transport activity [12]. Thus this may suggest that the common ancestor of plant aquaporins had lacked the ability to transport glycerol and later on NIPs had acquired this transport activity during evolution to compensate for the absence of GLPs in plants [17]. By reconstructing phylogenetic trees, Zardoya et al. [17] suggested that NIPs might originate from bacteria at the origin of plants through a single horizontal gene transfer event. Zardoya [9] confirmed the functional recruitment of NIPs to glycerol transport, however the gene horizontal transfer origination of NIPs could not be effectively recovered because of the general lack of resolution of deeper nodes.

The *Arabidopsis thaliana*, *Oryza sativa*, and *Physcomitrella patens *genomes encode 9, 13, and at least 5 NIP proteins respectively [3,4,7]. The occurrence and increase of NIP genes in plants indicate a wider range of function that may include a greater range in selectivity [12,18]. Functional analysis of NIPs indeed has revealed diversity in their transport substrates. The soybean nodulin26 protein not only transports water but also glycerol, formamide, malat, and NH_3 _[12,19,20]. In *Arabidopsis*, the AtNIP2;1 shows minimal water and glycerol transport, but displays transport of lactic acid with a preference under the anaerobic condition [21], whereas AtNIP5;1 is essential for efficient boron uptake and plant development under boron limitation [22]. Two NIPs (OsNIP2;1 and OsNIP2;2) in rice show a transport activity of larger solute silicic acid that enhances resistance of plants to biotic and abiotic stresses [23], whereas OsNIP1;1 and OsNIP3;1 do not; furthermore, the OsNIP2;1 can be permeable to water, urea, as well as boric acid, but not glycerol [24]. Therefore, the substrate specificity is NIP-dependent. Nonetheless, other unknown factors or structural features may also be involved in the process of efficient substrate recognition [24]. Very recently, some NIPs, including AtNIP1;1, AtNIP1;2, AtNIP5;1, AtNIP6;1, AtNIP7;1, OsNIP2;1, OsNIP2;2, OsNIP3;2, and LjNIP5;1, LjNIP6;1 were found to be responsible for the permeability to arsenite [25-28]. It can be proposed thus that arsenite transport through NIPs should be a conserved and ancient feature.

The molecular basis of aquaporins selectivity is pivotally due to two filters within the pore; the first one is formed by the conserved dual NPA motif, and the second one formed by a constriction region that is also called the ar/R (aromatic/arginine) filter [11,29,30]. However, it seems that the NPA motif is not crucial for [30,31], while the ar/R filter plays an important role in determining the substrate selectivity for the NIP subfamily [24]. The ar/R filter is located in the narrowest region on the extra-membrane mouth of the pore, and formed by four residues, one each from helix 2 (H2) and helix 5 (H5), as well as two residues from loop E (LE1 and LE2) [16]. It appears that the properties of the four residues making up the ar/R selectivity filter govern the substrate specificity of the pore [23], and are thought to be useful for predicting the function of the proteins [30]. Based on the ar/R regions of aquaporins, NIPs can be divided into three distinct groups [32]. NIP I proteins in *Arabidopsis *have been reported to transport water, glycerol, and lactic acid, whose ar/R region is characterized by Trp (W), Val (V)/Ile (I), Ala (A), and Arg (R). The consensus of the ar/R region for NIP II proteins, which are permeable to larger solutes than NIP I protein, is Thr (T)/Ala (A), Ala (A)/Ile (I)/Val (V), Gly (G)/Ala (A), and R [23,24]. OsNIP2;1 and OsNIP2;2 belong to the third group (NIP III) [24]. The ar/R region of NIP III consists of Gly (G), Ser (S), Gly (G), and Arg (R), forming a larger constriction size (≥6Å) compared with other NIP groups (≤5Å and 3.5Å), which allows solutes like silicic acid with a larger diameter (4.38Å) to permeate [24,31-33]. The critical role of the ar/R region is further demonstrated by the observations that substitution of Trp (W) with His (H) at the position H2 in LIMP2 abolished its glycerol transport when expressed in *Xenopus *oocytes [34]. Moreover, mutation at the Arg (R) residue in LE2 can cause human disease [35]. This residue is strictly conserved in NIPs, and thought to be important for providing hydrogen bonds for transport of water or glycerol molecules and to repel cations from the pore [36].

With a few of exceptions, the subcellular location of most members from the NIP subfamily is still uncertain. The archetype NIP, nodulin26 is located in the peribacteroid membrane (PBM) of soybean nodule cells [37]. The *Lotus japonicus *NIP gene, LIMP2 is also probably located in the PBM [38], whereas *Arabidopsis *AtNIP1;1 [28], AtNIP5;1 [22], rice NIP2 genes (OsNIP2;1 and OsNIP2;2) and the barley HvLsi1 are found to be localized on the plasma membrane [39-41]. In addition, it was found that the *Arabidopsis *AtNIP2;1 is predominantly expressed in young roots and is mainly located to the endoplasmic reticulum membrane [42]. Accordingly, the subcellular localization of each NIP protein may be diverse.

It has been reported that the activity of both plant and animal aquaporins may be regulated by phosphorylation [11]. The soybean NOD26 can be phosphorylated by a calcium-dependent protein kinase (CDPK) [37]; phosphorylation of NOD26 on Ser262 enhanced its water permeability [43]. The phosphorylated Ser262 of NOD26 is conserved in most but not all NIPs from *Arabidopsis *[44]. Experiments with the C-terminal extension of the *Arabidopsis *AtNIP7;1 showed that this gene could be phosphorylated by activated AtMPK4 in vitro [16]. Mitani et al. [24] however found no evidence for the involvement of phosphorylation in the regulation of OsNIP2;1, because neither okadaic acid nor K252a affected the transport activity of this protein for silicic acid in oocytes.

Although NIP proteins exhibit a similar three-dimensional structure [32], functional divergence has extensively occurred among NIP groups. It is therefore intriguing to further investigate the evolutionary mechanisms driving the functional diversification of the NIP groups at the nucleotide and amino acid sequence level respectively. Here we showed that strong positive selection had occurred after gene duplication and/or speciation. Functional divergence analysis provided convincing evidence for shifted evolutionary rate and/or rapid changes of amino acid properties between NIP groups, and identified some critical amino acid sites that are thought to be significantly functional divergence related. Further, we investigated the expression pattern and the *cis*-acting regulatory element organization in plant *NIPs *also.

## Results and discussion

### Phylogenetic and sequence character analysis of the plant NIP subfamily

On the basis of sequence similarity and the conserved MIP domain, we have identified 6, 8, 9, 11, 6, and 7 *NIP *genes from the *Vitis vinifera*, *Populus trichocarpa*, *Sorghum bicolor*, *Glycine max*, *Cucumis sativus*, and *Pinus taeda *genomes respectively (Additional file 1). Danielson and Johanson [7] revealed that the moss *Physcomitrella patens *encodes at least five NIP proteins, of which PpNIP6;1 is partial and excluded from the phylogenetic analysis. Furthermore, although *Chlamydomonas reinhardtii *might lack NIP proteins [45], one NIP homolog was identified from the green alga *Ostreococcus lucimarinus *in JGI (protein ID 25291), indicating that the NIP subfamily would be more ancient.

**Figure 1:**
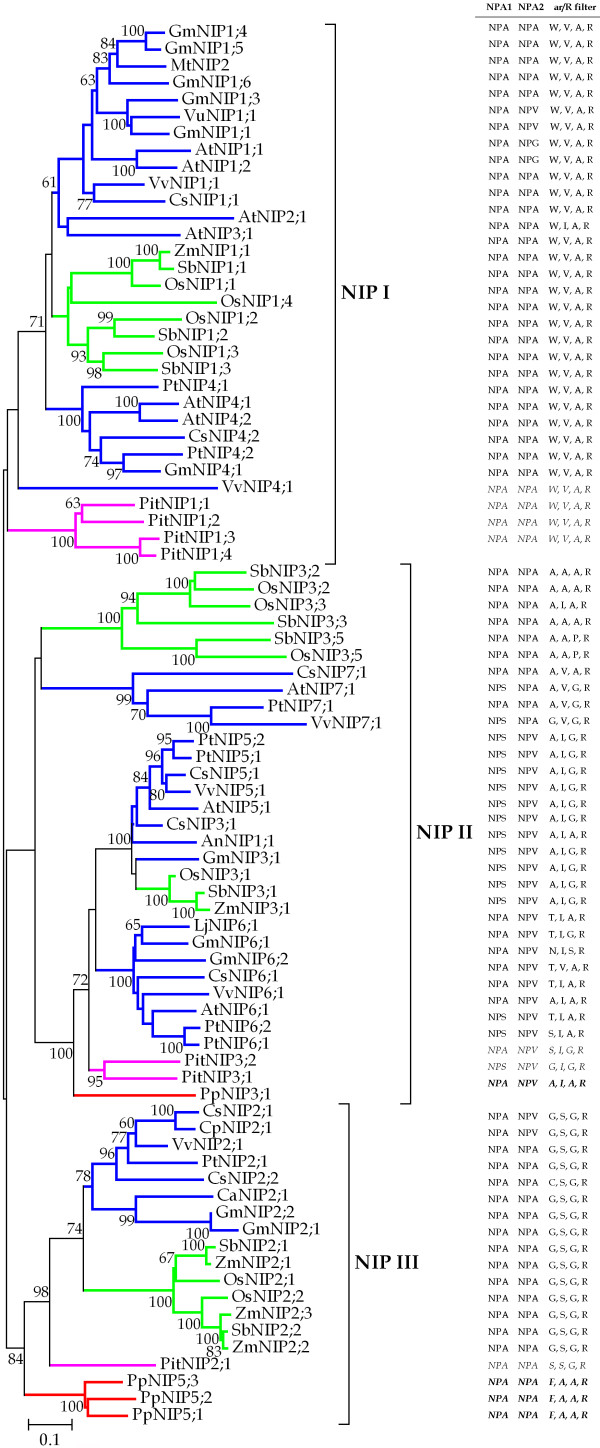
**Phylogenetic tree of NIP proteins in plants**. The *number *beside the branches represents bootstrap values ≥ 60% based on 1000 resamplings. The scale is in amino acid substitutions per site. The two NPA motifs and the four residues making up the ar/R filter are indicated in the figure. To identify the species of origin for each *NIP *gene, a species acronym is included before the gene name: An, *Atriplex nummularia*; At, *Arabidopsis thaliana*; Ca, *Cicer arietinum*; Cp, *Cucurbita pepo*; Cs, *Cucumis sativus*; Gm, *Glycine max*; Lj, *Lotus japonicus*; Mt, *Medicago truncatula*; Os, *Oryza sativa*; Pit, *Pinus taeda*; Pp, *Physcomitrella patens*; Pt, *Populus trichocarpa*; Sb, *Sorghum bicolor*; Vu, *Vigna unguiculata*; Vv, *Vitis vinifera*; Zm, *Zea mays*.

More than eighty NIP sequences were collected, aligned, and used to reconstruct phylogenetic trees. Figure 1 shows that plant NIPs are clearly divided into three groups, which supports the classification of NIPs based on the ar/R region [24,32]. It is apparent that each NIP group possesses at least one NIP protein each from *P. taeda *and *P. patens *(Figure 1), with the exception of the NIP I group where no *P. patens *NIPs are represented thus far, suggesting that the most recent common ancestor (MRCA) of *P. patens *and higher plants should have three ancestral *NIP *genes corresponding to the 3 groups. The diversification of the three ancient progenitor *NIPs *should have occurred before the emergence of the moss *P. patens *but after the green alga *O. lucimarinus*.

The diverse NIPs should have arisen from three ancestral genes, and subsequently species-specific expansion of this subfamily has occurred to a great extent. For example, three *P. patens *NIP5 (PpNIP5;1 – PpNIP5;3) and four *P. taeda *NIP1 isoforms (PitNIP1;1 – PitNIP1;4) were tightly clustered together in the NIP III and NIP I group respectively (Figure 1). Within each group, the monocot and dicot NIPs have formed distinct clades, indicating that the extensive proliferation of NIPs should have occurred after the monocot-dicot split. This proliferation may be attributed to the duplication of individual genes, or even the entire genome. The rice and *Arabidopsis *genomes have undergone two and three rounds of whole genome duplications (WGD) respectively [46,47], which may be responsible for the expansion of the NIP subfamily in the two model plants. We searched against the segmentally duplicated chromosome segments identified based on the TIGR rice (http://rice.plantbiology.msu.edu/segmental_dup/) and *Arabidopsis *(http://www.tigr.org/tdb/e2k1/ath1/Arabidopsis_genome_duplication.shtml) genome annotation release version 5.0 respectively. As expected, three gene pairs including *OsNIP1;1*/*OsNIP1;4*, *OsNIP1;2*/*OsNIP1;3*, and *OsNIP2;1*/*OsNIP2;2 *were found to be located into chromosomal regions that were supposed to have undergone large-scale segmental duplications. Moreover, three genes (*OsNIP3;2*, *OsNIP3;3*, and *OsNIP3;5*) were arranged as tandem on chromosome 8. As observed in rice, we also found evidence for segmental duplication (*AtNIP3;1*) and tandem duplications (*AtNIP1;1*/*AtNIP1;2*, and *AtNIP4;1*/*AtNIP4;2*) in *Arabidopsis*. The duplicated copy for the *AtNIP3;1 *gene however should have lost during evolution in that only one copy of it was found in the corresponding segmental duplicated regions. Thus, the explosive expansion of rice and *Arabidopsis NIPs *would attribute primarily to the segmental duplications and tandem duplications.

Furthermore, the exon/intron lengths and gene structure for each *NIP *gene were examined (Additional file 1), since the exon/intron structures were terribly important for investigating the evolution of any gene family. It was found that most of the *NIP *genes possess four or five exons, and only a few of them have 2, 3, or 6 exons. Moreover, we observed that with one exception (*OsNIP1;2*/*OsNIP1;3*), the gene pairs predicted to be produced by duplications showed similar gene structures, even though the lengths of introns might be different. For example, both *AtNIP4;1 *and *AtNIP4;2 *have 5 exons, and the length is the same for each of the five exons too. However, the lengths of the four introns vary extensively (Additional file 1). The similar cases were also found in the *OsNIP2;1*/*OsNIP2;2*, *OsNIP1;1*/*OsNIP1;4 *gene pairs, and in the *OsNIP3;2*/*OsNIP3;3*/*OsNIP3;5 *gene cluster. Although *OsNIP1;3 *lacks one exon than *OsNIP1;2*, the length of the second exon of the former gene is nearly equal to the total length of the second and third exons of the latter one. Besides, the lengths of the other three exons are similar to each other too (Additional file 1). It could be thus inferred that *OsNIP1;2 *should have independently gained an extra intron during evolution, because almost all NIP I genes in monocot plants possess four exons.

The two major constriction filters corresponding to the two NPA motifs and the ar/R filter were shown in Figure 1. It was observed that NIP proteins often have unorthodox NPA motifs, which is different from other MIPs. In the NIP subfamily, the first and second NPA motifs are always replaced by NPS and NPV (NPG), respectively (Figure 1, and Additional file 1). In the NIP I and III groups, the first NPA motif is highly invariant, and the second NPA motif is also conserved besides several exceptions. However, more complicated NPA patterns were present in the NIP II group. There are two types of NPA motifs each in the first (NPA and NPS) and second (NPA and NPV) positions. The monocot NIP3 and dicot NIP7 proteins possess conserved NPA in the second position, while it is replaced by NPV in other NIP II proteins (Figure 1). Wallace and Roberts [31] demonstrated that in AtNIP6;1 substitution of Ala for Val in the NPA2 region did not alter its transport selectivity, indicating that the NPA motifs should have little effect on determining the transport specificity for this protein.

The ar/R region is the second constriction filter. It is obvious that this region is highly subgroup specific for the NIP I and III groups. With one exception (AtNIP3;1), the ar/R region is characterized by W, V, A, R for the NIP I group. The hydrophobic residues W, V, and A, provide a non-polar surface that hydrophobically interacts with the hydrocarbon skeleton of glycerol, whereas the R residue creates a hydrogen bonding with the hydroxyls of glycerol, which facilitates the transit of glycerol and water molecules across the central water channel [32]. In NIP III, monocot and dicot NIPs possess a specific ar/R filter consisting of G, S, G, and R. The tiny character of G and S residues insures a wider aperture for the ar/R filter [32]. Nonetheless, there are some exceptions. Compared with CsNIP2;1, CsNIP2;2 possesses a different ar/R filter where the tiny Gly (G) residue is replace by the bulky Cys (C), and this ar/R filter may offer a narrower aperture than the former gene, indicating that the two genes may perform different transporter functions. The similar case was also found in PitNIP2;1, where the slight larger Ser (S) residue substitutes for the first Gly (G). The moss *P. patens *PpNIP5s have a unique combination of amino acids at the ar/R filter (Figure 1, see also [7]), where the four residues (F, A, A, and R) constitute a mediate aperture that should be larger than the NIP I protein, but narrower than the NIP III protein. A considerable variation in the ar/R region exists in NIP II proteins. The size and hydrophilicity of the four residues are different from each other, suggesting that members belonging to this group should function in different ways. Notwithstanding, the NPA motifs (NPA/NPV) and the ar/R filter (A, I, A, R) in the moss PpNIP3;1 is identical to AtNIP6;1, suggesting that these genes had the signatures before the split of bryophytes and vascular plants [7]. However, whether PpNIP3;1 functions as a boric acid transporter like AtNIP6;1 [48] needs further experimental investigation.

In addition, we found that except for the OsNIP3;2/OsNIP3;3/OsNIP3;5 gene cluster, other putative gene duplicates in rice and *Arabidopsis *possess the same NPA motifs and the ar/R signature (Additional file 1). The AtNIP4s (4;1 and 4;2) have the similar gene structure and the two constriction filters, suggesting that the two genes should perform same or similar functions in transporting water and glycerol, etc. In rice, both OsNIP2;1 and OsNIP2;2 were demonstrated to be permeable to the larger solute silicic acid [39,40], which supports our speculation on the AtNIP4s. Given that the ar/R regions in OsNIP3;2, OsNIP3;3 and OsNIP3;5 are clearly differentiated, we speculated that functional divergence should have occurred among them. Moreover, we found that the putative orthologs in the NIP I and III groups have the same (or with a slight modification) NPA motifs and the ar/R signature, whereas the orthologs in the NIP II group are different from each other (Figure 1). It was worth to note that the corresponding motifs in the green algae *O. lucimarinus*, are NPS/NAA, and Y, L, G, R respectively. If this is not the result of sequencing error, it can be inferred that this gene should play a quite different role from other NIPs in moss and higher plants.

In *Arabidopsis*, as much as seven NIP groups can be defined [3], whereas there are only three groups in rice [4,49]. This is a consequence of the large variation in NIP sequence divergence in dicot species [3]. It is worth noting that there is no *Arabidopsis *NIPs classified into the NIP III group, although the NIP2 genes of some dicots such as poplar, grape, and cucumber belong to this group (Figure 1). This suggests that *Arabidopsis *should have lost its counterpart in this group during evolution. Alternatively, it is likely that extensive sequence and structural variation should have occurred in *Arabidopsis AtNIP2;1*, leading to its functional divergence from other plant NIP2 genes. Accordingly we examined the gene structure and ar/R filter for this gene, and found that *AtNIP2;1 *possesses four exons and its ar/R filter is W, V, A, R, which is quite different from other NIP2 genes that always have five exons and whose ar/R filter is composed of G, S, G, R (see Additional file 1).

### Detection of positive selection signal

The multiple alignments of NIP protein sequences were shown in Additional file 2. The estimation of positive selection was based on the corresponding nucleotide ML tree (Additional file 3), where the sequences in partial and whose length are less than 240 codons were excluded so as to avoid possible analysis biases. To test for variable ω ratios among lineages, we conducted the likelihood ratio test (LRT) to compare the two extreme models: the one-ratio model that assumes a unique rate ratio for all branches, and the free-ratio model that assumes an independent ω ratio for each branch [50,51]. The log likelihood value under the one-ratio model is *-*23356.6, while the value is *-*23007.7 for the free-ratio model. Twice the log likelihood difference, 2Δℓ = 697.8, is strongly statistically significant, revealing a heterogeneous selective pressure among lineages. It is obvious that some branches of the NIP phylogeny include some internal branches having ω > 1, showing strong evidence for adaptive evolution. Moreover, we observed that the branches that were detected to be under positive selection should correspond to gene duplication or speciation events (see Additional file 3), which might act as a major evolutionary force driving the divergence of NIP functions.

The divergence of family members may involve positive selection, as indicated by many typical studies [52]. In a gene family, the fate of new genes produced by duplication would either evolve a new function under positive selection, or be lost during evolution [53]. Plants have evolved more abundant aquaporins with multifunctions, which may reflect the need for plants to better adapt external environmental conditions. Evidence for adaptive evolution thus clearly imply that functional diversification of the *NIPs *represents an evolutionary advantage for undergoing ecological adaptation to local environment [11]. It thus appears that continued positive selection should have acted on the *NIP *subfamily during evolution and this selection would be remarkably significant.

### Functional divergence analysis (FDA) of plant NIP proteins

Two types of functional divergence (Type-I and Type-II) between gene clusters of the NIP subfamily were estimated by posterior analysis using DIVERGE2 that evaluates shifted evolutionary rate and altered amino acid property after gene duplication [54,55]. The advantage of these methods is that they use amino acid sequences, and thereby is not sensitive to saturation of synonymous sites. The NIP subfamily consists of three major groups (NIP I, II, and III) ([24,32]; see also Figure 1). However, the results of positive selection analysis and primary FDA support the classification of NIP IIA and NIP IIB as two distinct groups (see Additional file 3). Thus, herein four gene clusters of interests were used as input for the DIVERGE2 analysis. As expected, the null hypothesis (no functional divergence) could be strongly rejected in that the coefficients of Type-I functional divergence (*θ*_I_) between NIP groups were statistically significant (*p *< 0.01; Table 1), indicating that significant amino acid site-specific selective constraints operate on different types of NIP members leading to a subgroup-specific functional evolution after their diversification. Further, a functional distance analysis was conducted. We estimated the functional branch length (*b*_F_) for each group by employing the least-squares method [54], and found that the level of altered selective constraints of group genes, measured by this index, followed *b*_F _(II, 0.48) > *b*_F _(IV, 0.32) > *b*_F _(I, 0.19) > *b*_F_(III, 0.12), suggesting that the NIP II group should be significantly divergent in function from other groups. On the other hand, it was found that the coefficients of Type-II functional divergence (*θ*_II_) between I/II, I/III, and I/IV were insignificant (*p *> 0.05). Nevertheless, we found evidences for Type-II functional divergence between three group pairs including II/III, II/IV, and III/IV, indicative of a radical shift of amino acid property [55].

**Table 1:** Functional divergence between groups of the plant NIP subfamily

Group1	Group2	Type-I					Type-II	
		
		*θ*_I _± S.E.	LRT	*p*	*Q*_*k *_> 0.8	*Q*_*k *_> 0.9	*θ*_II _± S.E.	*Q*_*k *_> 0.8
NIP I	II	0.449 ± 0.063	51.3	<0.01	17	8	-0.085 ± 0.163	0
NIP I	III	0.190 ± 0.068	7.7	<0.01	2	0	-0.105 ± 0.175	0
NIP I	IV	0.429 ± 0.087	24.5	<0.01	7	3	0.004 ± 0.178	0
NIP II	III	0.486 ± 0.068	50.6	<0.01	25	11	0.106 ± 0.112	2
NIP II	IV	0.534 ± 0.098	29.4	<0.01	13	3	0.131 ± 0.115	2
NIP III	IV	0.286 ± 0.092	9.7	<0.01	1	1	0.221 ± 0.119	7

*Note: θ*_I _and *θ*_II_, the coefficients of Type-I and Type-II functional divergence between two gene clusters; LRT, Likelihood Ratio Statistic; *Q*_*k*_, posterior probability. Large *Q*_*k *_value indicates a high possibility that the functional constraint (or the evolutionary rate) or the physicochemical properties of a given amino acid site is different between two clusters.

To identify critical amino acid sites that may be responsible for functional divergence between NIP groups, the posterior probability (*Q*_*k*_) of divergence was determined for each site. According to the definition, large *Q*_*k *_indicates a high possibility that the evolutionary rate or amino acid physiochemical property of a site is different between two clusters. DIVERGE2 thus identified some critical amino acid sites (CAASs) that are highly relevant to functional divergence (see Additional file 4, and Table 1). In order to extensively reduce positive false, *Q*_*k *_> 0.8 was empirically used as cutoff to identify the Type-I and Type-II functional divergence-related residues between gene clusters. The results showed that more than 13 CAASs were supposed to be responsible for the functional divergence between NIP II and I, III, IV respectively; whereas there were only two, seven, and one CAAS with *Q*_*k *_> 0.8 identified between I/III, I/IV, and III/IV respectively. Interestingly, in contrast to the Type-I functional divergence, two, two, and seven Type-II related CAASs were identified for the II/III, II/IV, and III/IV pairs respectively (Table 1). Compared with only one CAAS for the Type-I functional divergence, there were seven predicted sites for the Type-II functional divergence between III/IV, indicating that the functional divergence between the two group genes was mainly attributed to the rapid changes of amino acid physiochemical property, and secondly to the shifted evolutionary rate. The contrary cases were found for the II/I, II/III, and II/IV pairs. These observations indicated that site-specific shift of evolutionary rate and changes of amino acid property should not uniformly act on the NIP subfamily members during long periods time of evolution. The relative importance of Type-I and Type-II functional divergence may be associated with specific functional classes of this protein family. However, the degree of functional divergence between NIP I/III was not remarkably significant, because there were only two CAASs with posterior probability >0.8. This suggests that genes belonging to these two groups might perform similar functions in some aspects [25,27,28].

These CAASs identified by DIVERGE2 were mapped onto the alignments of protein sequences (Additional file 4). We found that these CAASs were mainly located in four transmembrane regions (TMs 1, 2, 5, and 6), and only a few of them fell into the intra- or extra-cellular loops. We take the II/III pair as an example. There were 25 and 2 CAASs obtained for the two types of functional divergence respectively. Among the 27 predicted CAASs, there were 10 sites located in the loop regions, while 17 sites were in the TMs, with particular abundance in TM6. The similar cases were also found in other group pairs (Additional file 4). Given the observation that no possible CAAS was identified in TM4, it indicated that this region should be more conserved during evolution, and play important roles in maintaining the fundamental function of NIP aquaporins. In reality, conserved amino acids located in the helices 4 and 6 are essential to maintain the tetrameric structure of aquaporins [56].

We observed that among the predicted CAASs, two sites for the I/II pair, and one each for the II/III, and II/IV pairs corresponded to the first and/or third residues in the ar/R region (Additional file 4), suggesting that selective forces should have worked on the two residues of the ar/R filter, thereby leading to the diversity of substrate selectivity for different NIP proteins. In NIP I, the ar/R residues include W, V/I, A, and R, while these positions are occupied by A/G/T/S/N, A/I/V, G/A, and R for the NIP II group. The two identified CAASs in the I/II pair correspond to the residues in the H2 and LE1 positions, which involve the changes of the size and hydrophilicity of the corresponding residues. For example, the bulky Trp (W) in NIP I was replaced by the tiny A/S/T/N in NIP II; the hydrophilic G/S substituted for the hydrophibic A. These changes play a pivotal role in determining the substrate specificity. It has been reported that NIP I proteins can permeate small solutes such as water and glycerol, whereas NIP II proteins show no measurable water permeability but transport glycerol, formamide, as well as some larger uncharged solutes than NIP I protein. Experimental evidences showed that the substitution of a Trp (W) residue for Ala (A) at the H2 position of the ar/R tetrad of AtNIP6;1 results in enhanced water-transport activity, but the permeability of the mutant protein to urea, similar to the NIP I protein soybean nodulin 26, becomes more restrictive [31]. Using site-directed mutagenesis method, Wallace et al. [34] demonstrated that the residue in the H2 position should be a major determinant of glycerol selectivity.

In the II/III pair, the third residue (LE1) of the ar/R filter is predicted to be functional divergence related. This position is invariant Gly (G) in NIP III proteins, whereas the same position in NIP II contains several amino acids (glycine, alanine, or serine) with different properties, such as the nonpolar Ala (A). The size of the four residues (G, S, G, and R) in NIP III is smaller compared to other groups of NIPs, which form a larger constriction size (≥6Å) and allow for passage of much larger solutes [24]. However, NIP III proteins cannot be permeable to glycerol [57], although the molecular size of glycerol is smaller than that of silicic acid. This suggests that other unknown factors may also be involved in the substrate specificity [24].

To date, the monocot NIP3s (defined as NIP IV in this study) were included in the NIP II group. The present study however provided statistical evidence that monocot NIP3s should have largely diverged from other NIP II proteins in function, and fifteen CAASs including the H2 residue of the ar/R region were predicted to be responsible for the functional divergence (Table 1 and Additional file 4). In NIP IV, the residue in H2 is highly conserved to Ala (A). In contrast, several types of amino acids, such as alanine, asparagines, threonine, and serine, occupy the corresponding position in the NIP II group. However, whether this residue position is determinant for functional differentiation between NIP II and IV groups remains unknown.

Using more than 150 MIP proteins, Froger et al. [58] identified five positions (P1-P5) where the physicochemical properties of the corresponding amino acids are drastically different in aquaporins and glycerol permeases. Of the five positions, P4 and P5 correspond to two consecutive amino acids located in the sixth transmembrane segment (TM6). Lagrée et al. [59] demonstrated that mutations of YW to PL at the two positions totally converted the selectivity of the channel from water to glycerol. Thus, it seems that positions P4P5 play a crucial role in transport specificity. The five positions were designated in Additional file 4. We found that one position (P5) each for the I/II and II/III pairs was predicted to be highly functional divergence related, suggesting that the corresponding position should have acted as a determinant factor in subgroup-specific substrate transport.

Moreover, we detected some "hotspot" amino acid sites that are highly contributed to the functional divergence between different NIP subgroups. For example, there are three (I54, Q72, and A245) and four (P44, S85, Q125, and G242) such positions identified in OsNIP1;1 and OsNIP2;1 respectively (Additional file 4). However, because plant NIPs are comparatively less studied, the functional importance of the CAASs identified firstly needs to be further experimentally examined.

### Expression analysis of NIP genes

**Figure 2:**
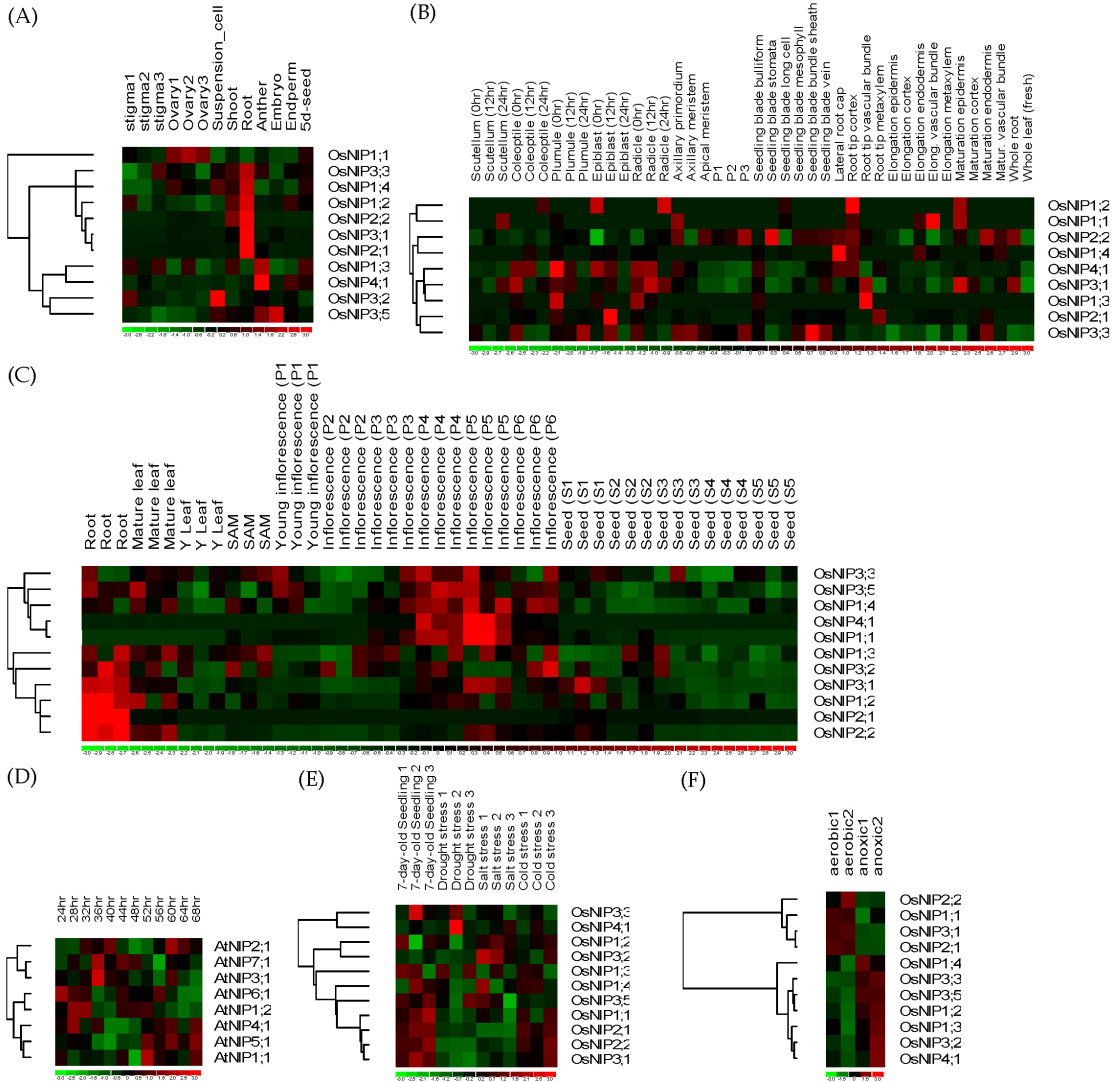
Gene expression patterns of rice and/or *Arabidopsis NIP *genes in nine tissues (A), forty cell types (B), during the reproductive development (C), under constant white light (D), in response to drought, salt and cold (E), as well as anoxic (F) treatments.

The transcriptional patterns of rice *NIP *genes in nine tissues and forty cell types were investigated. It is observed that *OsNIPs *are unevenly expressed in the examined tissues, and exhibit a clearly tissue-specific expression pattern. *OsNIP2;1*, *OsNIP3;1*, and *OsNIP2;2 *are predominantly expressed in root (Figure 2A); their Specificity Measure (SPM) values are 0.994, 0.991, and 0.864 respectively. Similarly, relative to other tissues, *OsNIP4;1 *and *OsNIP3;2 *have a much stronger expression level in anther (SPM = 0.828) and suspension cell (SPM = 0.810) respectively, suggesting that they should play specific roles in the corresponding tissues. The similar cases were also found in *Arabidopsis *(Additional file 5). Consistent with the above postulation, Ma et al. [39] demonstrated that *OsNIP2;1 *was responsible for the uptake of silicic acid from soil. The poplar *PtNIP1;1 *was specifically expressed in the suspensor ligament of the embryo, and played important roles in the corresponding process [60]. Further, the microarray data for rice *NIP *genes in forty cell types were analyzed. It is obvious that *OsNIPs *represent a cell-type-specific expression pattern (Figure 2B), supporting the idea that NIP transport activities may be prevalent in a more defined set of cells in the plant [16]. This result was further validated by the analysis of *Arabidopsis AtNIPs *in the root cell-types (Additional file 6).

We further analyzed the expression of *OsNIPs *during the reproductive development, and found that the *OsNIPs *could be classified in two groups of expression pattern (Figure 2C). Five *OsNIPs *(3;3, 3;5, 1;4, 4;1, and 1;1) that are largely expressed in the inflorescence stage, represent the first group, while the other *OsNIPs *that appear to be root specific, form the second one. Therefore, the expression of *NIPs *would be developmental-related. Coincidently, it was found that *PsNIP1;1 *was expressed in the developing seed coats of pea seeds in a developmentally specific manner [61].

The expression of *AtNIPs *is generally low under the treatment of constant white light, but the responses of *AtNIPs *are different (Figure 2D). For example, *AtNIP3;1 *exhibits a higher expression level under 36 hrs constant lighting. Moreover, we observed that most of the *OsNIPs *expression is down-regulated under a variety of stresses, such as drought, salt, and cold (Figure 2E). However, *OsNIP4;1 *is highly regulated by drought. These results indicate that *NIPs *are not exception in responding to a diverse array of stress related signals. Weig et al. [62] found that the expression of *AtNIP1;1 *was severely down-regulated by the stresses drought, salinity, as well as hormone abscisic acid. *Arabidopsis *AtNIP2;1 is an anaerobic-induced lactic acid transporter that may play a role in adaptation to lactic fermentation under anaerobic stress [21], which promotes us to further examine the expression profiles of *OsNIP *genes under the aerobic and anoxic conditions [63]. Interestingly, we observed that in rice coleoptile *OsNIPs *could be clearly divided into two groups: the first group includes four genes (*OsNIPs *1;1, 2;1, 2;2, and 3;1), which are aerobic-dependent; the second group consists of the other seven *OsNIPs*, whose expression is regulated by anoxic stress (Figure 2F). Overall, the detailed study of *NIP *expression will refine our understanding of their physiological roles in plants.

### Regulatory elements for plant NIP genes

Transcription factors bind to corresponding TFBSs (transcription factor binding sites) upstream from genes of interest, and the profiles of *cis*-acting elements may thus provide information for understanding the regulatory mechanism of gene expression. A computational tool PlantCARE [64] was adopted to identify putative TFBSs in the 1000 bp DNA sequence upstream of the translation initiation codon of *NIP *genes in rice, *S. bicolor*, grape and poplar.

Four types of *cis*-elements were found to be significantly abundant in the promoter region of plant *NIP *genes (Additional file 7). The first type of *cis*-element enriched in the promoter region is the light responsive elements, such as G-Box [65], GAG-motif [66], and Box 4 [67] etc. G-Box is the most abundant *cis*-element in rice. All but two (*OsNIP3;3 *and *OsNIP4;1*) have at least one copy of this element, whereas the Box 4 element appears to be more abundant in grape and poplar. Plant hormone responsive elements, such as ABRE [68], P-box [69], as well as the TCA-element [70], constitute the second class. It seems that ABRE is the most abundant hormone-related *cis*-element in rice, suggestive of the regulation of the expression of some *OsNIPs *by abscisic acid (ABA); whereas no such element has been detected in grape and poplar. However, the *AtNIP1;1 *expression was remarkably affected by ABA [62]. In contrast, the salicylic acid responsive TCA-element is found frequently in grape and poplar. These observations suggest that monocot and dicot plant *NIPs *should be significantly regulated by different types of hormones. The third class of *cis*-element in abundance consists of elements in response to external environmental stresses (Additional file 7). Guenther et al. [43] demonstrated that the phosphorylation of soybean nodulin 26 was enhanced by osmotic signals (both drought and salt stress). We observed that nearly All *NIPs *examined here appear to contain the ARE [71] and MBS [72] elements. ARE is an element involved in anaerobic induction [71]. In rice coleoptile, *OsNIPs *are classified in two distinct expression patterns (Figure 2F). We speculated therefore that the anaerobic regulation of *OsNIPs *expression should be tissue or developmental-stage dependent. The drought responsive element MBS [72] is also enriched in the promoter. With a few exceptions, *OsNIPs *contain more than two copies of this element (Additional file 7). Circadian that is involved in circadian control [73] is the fourth type of *cis*-element found abundant in the promoters of monocot *NIP *genes. PlantCARE [64] identified one circadian in the rice silicic acid transporter OsNIP2;1 [39], which may be responsible for its distinct diurnal expression pattern [74]. The presence of a diverse of *cis*-elements in the upstream regions of *NIPs *indicates that plant *NIPs *may function in a wider range of ways.

In addition, *NIP *membership-specific *cis*-elements have been observed. For example, in rice, *OsNIP2;1 *and *OsNIP3;2 *each possesses a putative LTR motif that is response to low temperature [75,76]. AC-I, a *cis*-element conferring enhanced xylem expression [77,78], is specifically present in *OsNIP1;1*. Furthermore, orthologous *NIPs *in monocot and dicot plants have different *cis*-element organization as well (Additional file 7). These results suggest that plant *NIPs *should have evolved some specific regulatory elements, and thereby leading to the differentiation of expression patterns.

## Conclusion

Plant NIP subfamily is more ancient, and their diversification can be placed at the time before the emergence of the moss *P. patens*. As many typical gene families, *NIPs *have experienced strong positive selection during evolution. Consistently, the amino acid level analysis suggests that functional divergence has occurred between plant NIP proteins, and identified the critical amino acid sites involved in this divergence for further investigation. Rice and *Arabidopsis NIPs *exhibit distinct expression pattern. The survey of upstream elements reveals four major classes of *ci*s-elements in the promoter region of *NIPs *and their distinct organization pattern is interpreted to reflect their varying participation in gene expression regulation. The rapid proliferation and functional diversification of plant NIPs is argued to have partially attributed to the need for plants to better adapt to external different environments. These findings provide new insights into understanding the evolutionary mechanisms of NIP proteins and their functional diversification.

## Methods

### Sequence data

*O. sativa*, *A. thaliana*, *Z. Mays*, and *P. patens *NIP sequences were downloaded from the GenBank and JGI databases according to the published literatures [3,7,49,79]. The NIPs were used as query to search against the *V. vinifera*, *P. trichocarpa*, *S. bicolor*, *G. max*, *C. sativus*, and *P. taeda *genomes using the BLASTP and TBLASTN programs respectively. Other sequences including the *Cicer arietinum *CaNIP (AN: CAG34223), and *Cucurbita pepo *CpNIP (AN: CAD67694) were collected from literature [32]. Programs InterProScan [80] and ConPred II [81] were employed to detect the conserved MIP domain and predict the putative transmembrane regions respectively.

### Multiple sequence alignment and phylogenetic tree reconstruction

Plant NIP protein sequences were aligned using the program L-INS-i implemented in MAFFT v6.6 [82], with the parameters: Scoring matrix for amino acid sequences, BLOSUM62; Gap opening penalty, 2.0; and Gap extension penalty, 0.2. The resulting protein alignment was subsequently employed to generate the codon-alignment of corresponding coding DNA sequences using a custom PERL script. Maximum likelihood (ML) phylogenies were reconstructed with PHYML v2.4 [83]. The programs PROTTEST [84] and ModelGenerator [85] were utilized to determine the best model for each ML analysis. Here the JTT+I+G model for the protein alignment and the HKY+I+G+F model for the codon alignment were determined respectively. The reliability of interior branches was assessed with 500 bootstrap resamplings. Phylogenetic trees were displayed using MEGA v4.0 [86].

### Test of positive selection

The CODEML program implemented in the PAML v4.0 software package [51] was utilized to test the hypothesis of positive selection in the *NIP *subfamily during evolution. To test for heterogeneous selective pressure among lineages [50], models of variable ω ratios among lineages were fitted by ML to the *NIP *sequence alignment. The ratio of nonsynonymous-to-synonymous for each branch under two models (one-ratio and free-ratio for branches) was calculated, and the two models were compared using the LRT test to see whether the ω ratios are different among lineages; that is, positive selection is indicated if the free-ratio model that allows for selection is significantly better than the one-ratio model (no selection) in the LRT analysis.

### Estimation of functional divergence

The software DIVERGE2 [55] was used to detect functional divergence between members of the plant NIP protein subfamily, where 4 gene clusters of interests were selected. The coefficients of Type-I and Type-II functional divergence *θ*_I _and *θ*_II _between any two NIP groups were calculated. If *θ*_I _or *θ*_II _is significantly greater than 0, it means site-specific altered selective constraints or a radical shift of amino acid physiochemical property after gene duplication [54,55]. Moreover, a site-specific posterior analysis was used to predict amino acid residues that were crucial for functional divergence.

### Investigation of transcription patterns

Gene expression microarray datasets (GSE7951, GSE13161, GSE6893, GSE6908, and GSE6901 for rice; GSE680, GSE7641, and GSE8365 for *Arabidopsis*) were downloaded from the GEO database in NCBI. The microarray data of rice include the analysis of gene expression profiles in nine tissues [87] and forty cell types; during reproductive development; seven-day-old seedlings under drought, salt, and cold stress treatments [88]; and rice coleoptile under the aerobic and anoxic conditions [63]. In *Arabidopsis*, the transcript profiles of *NIP *genes in root cell-types after treatment with salt [89], under constant white light treatment [90], and during the whole plant life cycle, were investigated as well. Program dChip 2008 (5/8/08) [91] was used to perform the cluster analysis and display the expression patterns of rice and *Arabidopsis NIP *genes using microarray data as input. The GEPS software [92] was employed to quantitatively analyze the expression pattern of *NIP *genes. Specificity Measure (SPM) was used to define the tissue-specific expression pattern of a gene, which may be useful for further understanding its physiological behaviors [92].

### Analysis of cis-acting regulatory elements

1000 bp of nucleotide sequences upstream of the translation initiation codon for each NIP gene in four species (rice, *S. bicolor*, grape, and poplar) was extracted, which were used for the TFBSs analysis. At present, no full-length cDNA sequences for grape were available. In order to facilitate comparison between species, the sequences upstream of translation initiation codon rather than transcription start site were used to screen possible *cis*-acting regulatory elements. The software PlantCARE [64] was utilized to determine putative plant-specific TFBSs in a given DNA sequence. To avoid biases in analysis, only TFBSs whose matrix score is not less than 6 were considered further.

## Authors' contributions

QL and ZZ conceived and designed the experiments. QL, HW, and YF performed the experiments and analyzed the data. ZHZ identified and annotated the exon/intron structures of the *NIP *genes in cucumber. JW facilitated the usage of the dChip program and performed the statistical analysis. QL wrote the paper. All authors read and approved the final manuscript.

## Supplementary Material

Additional file 1 **List of *NIP *genes in plants**. For each *NIP *gene, the information about the accession number, chromosomal localization, gene length, protein length, gene structure, the NPA motif, and the ar/R filter sequence were listed. Click here for file

Additional file 2 **The multiple alignments of plant NIP protein sequences**. These sequences were aligned using the program L-INS-i implemented in MAFFT v6.6. The alignments were shaded in the "Quantify Mode", and the residues were displayed in the "Difference Mode" with the "Diff/Consensus Line" style. Dots and "-" indicate similar residues and gaps on the alignment respectively. Click here for file

Additional file 3 **Phylogenetic tree reconstructed using plant *NIP *nucleotide sequences**. The *number *beside the branches represents bootstrap values ≥ 300 based on 500 resamplings. The scale bar shows total nucleotide distance. The *NIP *homologue in the green alga *Ostreococcus lucimarinus *(defined as *galgaNIP*) is used as outgroup sequence to root the tree. Branches with rates of numbers of nonsynonymous and synonymous substitutions >1, are indicated by red thick lines. Click here for file

Additional file 4 **Functional divergence significantly related amino acid site candidates**. A site-specific profile based on the posterior probability (*Q*_*k*_) was used to identify critical amino acid sites that were responsible for functional divergence between NIP subfamily members. According to the definition, large *Q*_*k *_indicates a high possibility that the functional constraint (or, the evolutionary rate) or the radical change of amino acid property of a site is different between two clusters. Dots indicate conserved residues with the first protein, such as OsNIP2-1. Amino acids (AAs) with *Q*_*k *_values 0.9>*Q*_*k*_>0.8 and *Q*_*k*_>0.9 are shaded with green and red color respectively. The predicted AAs for Type-II functional divergence are shown in purple. The six predicted transmembrane regions (TMs) are marked above the sequences. Arrows indicate the first and/or the third residue(s) in the ar/R region. The five positions identified by Froger et al. [58] are indicated by stars. (A) I/II; (B) I/III; (C) I/IV; (D) II/III; (E) II/IV; (F) III/IV. Click here for file

Additional file 5 **The expression patterns of *Arabidopsis AtNIPs *during the plant life cycle.** Click here for file

Additional file 6 **The diversity of expression profiles of *Arabidopsis AtNIP *genes in root cell-types after treatment with salt.** Click here for file

Additional file 7 **Analysis of *cis*-acting elements in the 1000 bp sequence upstream of the translation initiation codon in plant *NIP *genes.** Click here for file
