# Supplementary material for: Divergence in function and expression of the NOD26-like intrinsic proteins in plants
Source: BMC Genomics. 2009 Jul 15;10:313. doi: 10.1186/1471-2164-10-313 (PMC2726226; doi:10.1186/1471-2164-10-313)
Supplement: Additional file 1 — List of NIP genes in plants. For each NIP gene, the information about the accession number, chromosomal localization, gene length, protein length, gene structure, the NPA motif, and the ar/R filter sequence were listed. [file 1471-2164-10-313-S1.pdf]

Additional file 1

(A) List of *NIP* genes in plants

NIPs	Protein ID	Gene ID	Chromosome	Gene length	CDS length	AA length	Gene structure	cDNA	NPA motif1	NPA motif2	ar / R filter	Reference
OsNIP1;1	BAD27715	AP004070	2	2994	855	284		AK068806	NPA	NPA	W, V, A, R	1,3,4
OsNIP1;2	BAD73177	AP003105	1	3547	912	303			NPA	NPA	W, V, A, R	1,3
OsNIP1;3	AAV44140	AC135918	5	4553	861	286		AK062320	NPA	NPA	W, V, A, R	1,3
OsNIP1;4	BAD53665	AP003682	6	1113	822	273			NPA	NPA	W, V, A, R	1,3
**OsNIP2;1**	**BAD16128**	**AP005297**	**2**	**3609**	**897**	**298**		**AK069842**	**NPA**	**NPA**	**G, S, G, R**	**1-4,8,13**
**OsNIP2;2**	**BAD37471**	**AP003569**	**6**	**4567**	**897**	**298**		**AK112022**	**NPA**	**NPA**	**G, S, G, R**	**1,3,4,13**
OsNIP3;1	AAG13499	AC068924	10	1573	726	241			NPS	NPV	A, I, G, R	1,3,4
OsNIP3;2	BAC99758	AP005467	8	1366	918	305			NPA	NPA	A, A, A, R	1,3,8
OsNIP3;3	BAC65382	AP005467	8	1426	837	278			NPA	NPA	A, I, A, R	1,3
OsNIP3;5	BAC98553	AP004461	8	3021	852	283			NPA	NPA	A, A, P, R	1,3
OsNIP4;1	BAB61180	AP003219	1	1124	861	286		AK106825	NPA	NPA	C, G, G, R	1,3
ZmNIP1;1	AAK26750	AF326483	AC215509.2	1852	849	282		AF326483	NPA	NPA	W, V, A, R	5
ZmNIP2;1	AAK26751	AF326484	AC196472.2	3103	888	295		AF326484	NPA	NPA	G, S, G, R	5
ZmNIP2;2	AAK26752	AF326485	AC206502.3	3272	885	294		AF326485	NPA	NPA	G, S, G, R	5
ZmNIP2;3	AAK26849	AF342810	AC216219.3	3088	906	301		AF342810	NPA	NPA	G, S, G, R	5
ZmNIP3;1	AAK26753	AF326486	AC209805.3	2752	909	302		AF326486	NPS	NPV	A, I, G, R	5
AtNIP1;1	CAA16760	AL021711	4	1681	927	308			NPA	NPG	W, V, A, R	6,7,9
AtNIP1;2	CAA16748	AL021711	4	1968	885	294			NPA	NPG	W, V, A, R	6,7,9
AtNIP2;1	AAC26712	AC004077	2	1176	867	288			NPA	NPA	W, V, A, R	6,7,11,12
AtNIP3;1	AAG50717	AC079041	1	1124	810	269			NPA	NPA	W, I, A, R	6,7
AtNIP4;1	BAB10360	AB016873	5	2575	852	283			NPA	NPA	W, V, A, R	6,7
AtNIP4;2	BAB10361	AB016873	5	1281	852	283			NPA	NPA	W, V, A, R	6,7
AtNIP5;1	CAB39791	AL049488	4	2980	915	304			NPS	NPV	A, I, G, R	6-9
AtNIP6;1	AAF14664	AC011713	1	1375	918	305			NPA	NPV	A, I, A, R	6-8
AtNIP7;1	AAF30303	AC018907	3	1546	825	274			NPS	NPA	A, V, G, R	6,7,14

CpNIP2;1	CAD67694	AJ544830	——	——	867	288	——	AJ544830	NPA	NPV	G, S, G, R	
CaNIP2;1	CAG34223	AJ746344	——	——	822	273	——	AJ746344	NPA	NPA	G, S, G, R	
VuNIP1;1	ABS72446	EF514501	——	——	822	273	——	EF514501	NPA	NPV	W, V, A, R	
LjNIP6;1	ABY19374	EU294215	——	——	942	313	——	EU294215	NPA	NPV	T, I, A, R	8
MtNIP2	AAS48064	AC152446	——	2018	810	269		AY539750	NPA	NPA	W, V, A, R	10
AnNIP1;1	AAQ11826	AF544251	——	——	903	300	——	AF544251	NPS	NPV	A, I, A, R	
PpNIP3;1	94322	94322	Scaffold_258	1732	906	301			NPA	NPV	A, I, A, R	15
PpNIP5;1	115513	115513	Scaffold_13	1272	687	228			NPA	NPA	F, A, A, R	15
PpNIP5;2	186237	186237	Scaffold_91	1617	714	237			NPA	NPA	F, A, A, R	15
PpNIP5;3	179749	179749	Scaffold_37	2505	696	231			NPA	NPA	F, A, A, R	15
SbNIP1;1	4882657	5111844	4	2861	864	287			NPA	NPA	W, V, A, R	
SbNIP1;2	4804466	5033653	3	1091	816	271			NPA	NPA	W, V, A, R	
SbNIP1;3	5060249	5289436	9	3306	852	283			NPA	NPA	W, V, A, R	
SbNIP2;1	4757201	4986388	4	3781	894	297			NPA	NPA	G, S, G, R	
SbNIP2;2	149942	149942	10	4774	888	295			NPA	NPA	G, S, G, R	
SbNIP3;1	4742451	4971638	1	4668	906	301			NPS	NPV	A, I, G, R	
SbNIP3;2	5043039	5272226	7	1187	894	297			NPA	NPA	A, A, A, R	
SbNIP3;3	4840652	5069839	7	907	870	289			NPA	NPA	A, A, A, R	
SbNIP3;5	5043032	5272219	7	1078	894	297			NPA	NPA	A, A, P, R	
PtNIP2;1	577637	577637	XVII	3290	837	278			NPA	NPA	G, S, G, R	
PtNIP4;1	566501	566501	X	1433	792	263			NPA	NPA	W, V, A, R	
PtNIP4;2	756079	756079	II	2042	840	279			NPA	NPA	W, V, A, R	
PtNIP5;1	708017	708017	I	4931	900	299			NPS	NPV	A, I, G, R	
PtNIP5;2	823094	823094	XI	3914	903	300			NPS	NPV	A, I, G, R	
PtNIP6;1	757987	757987	III	2410	912	303			NPS	NPV	S, I, A, R	
PtNIP6;2	797136	797136	I	2848	912	303			NPS	NPV	T, I, A, R	
PtNIP7;1	803915	803915	VIII	2253	729	242			NPA	NPA	A, V, G, R	
GmNIP1;1	Gm0003x00182	—	Scaffold_3	4078	732	243			NPA	NPV	W, V, A, R	
GmNIP1;3	Gm0022x00351	—	Scaffold_22	3302	816	271			NPA	NPA	W, V, A, R	

GmNIP1;4	Gm0022x00350	—	Scaffold_22	2387	825	274			NPA	NPA	W, V, A, R	
GmNIP1;5	Gm0003x00181	—	Scaffold_3	2100	813	270			NPA	NPA	W, V, A, R	
GmNIP1;6	Gm0030x00284	—	Scaffold_30	2434	822	273			NPA	NPA	W, V, A, R	
GmNIP2;1	Gm0021x00508	—	Scaffold_21	5064	888	295			NPA	NPA	G, S, G, R	
GmNIP3;1	Gm0131x00049	—	Scaffold_131	5663	912	303			NPS	NPV	A, I, G, R	
GmNIP6;1	Gm0088x00395	—	Scaffold_88	3652	915	304			NPA	NPV	T, I, G, R	
GmNIP6;2	Gm0113x00121	—	Scaffold_113	4120	921	306			NPA	NPV	N, I, S, R	
PitNIP1;1	—	TA9262_3352	——	1290	819	272	——		NPA	NPA	W, V, A, R	
PitNIP1;2	—	TA21380_3352	——	1330	789	262	——		NPA	NPA	W, V, A, R	
PitNIP1;3	—	TA13914_3352	——	816	714	237	——		NPA	NPA	W, V, A, R	
PitNIP1;4	—	AY055751	——	1065	795	264	——		NPA	NPA	W, V, A, R	
PitNIP2;1	—	TA24411_3352	——	904	681	226	——		NPA	NPA	S, S, G, R	
PitNIP3;1	—	TA8714_3352	——	1752	885	294	——		NPS	NPV	G, I, G, R	
PitNIP3;2	—	TA16960_3352	——	1770	912	303	——		NPA	NPV	S, I, G, R	
VvNIP1;1	CAO48005	CU459298	10	1891	789	262			NPA	NPA	W, V, A, R	
VvNIP2;1	CAO15462	CU461115	Scaffold_2214	2268	738	245			NPA	NPA	G, S, G, R	
VvNIP4;1	CAO70192	CU459401	Scaffold_184	37269	741	246			NPA	NPA	W, V, A, R	
VvNIP5;1	CAO62847	CU459322	2	3306	897	298			NPS	NPV	A, I, G, R	
VvNIP6;1	CAO45476	CU459286	Scaffold_69	2268	1065	354			NPA	NPV	T, I, A, R	
VvNIP7;1	CAO71103	CU459219	5	1263	882	293			NPS	NPA	G, V, G, R	
CsNIP1;1	—	—	Scaffold000020	1518	831	276			NPA	NPA	W, V, A, R	
CsNIP2;1	—	—	Scaffold002257	4156	867	288			NPA	NPV	G, S, G, R	
CsNIP2;2	—	—	Scaffold001348	2898	786	261			NPA	NPA	C, S, G, R	
CsNIP4;2	—	—	Scaffold000444	3817	810	269			NPA	NPA	W, V, A, R	
CsNIP5;1	—	—	Scaffold000511	4801	897	298			NPS	NPV	A, I, G, R	
CsNIP6;1	—	—	Scaffold000151	2347	915	304			NPA	NPV	T, V, A, R	
CsNIP7;1	—	—	Scaffold000085	1595	603	200			NPA	NPA	A, V, A, R	
galgaNIP	XP_001420576	XM_001420539	12	949	813	270		XM_001420539	NPS	NAA	Y, L, G, R	

Note: The "—" indicates that no accession number was assigned to the corresponding genes in the GenBank database at present. "——" indicates that the corresponding data is

unavailable now. The predicted protein and nucleotide sequences of SbNIPs, PtNIPs, GmNIPs, and VvNIPs were downloaded from JGI (http://genome.jgi-psf.org). The *Cucumis sativus* NIPs were personally communicated with the Chinese Academy of Agricultural Sciences. To identify the species of origin for each NIP, a species acronym is included before the protein name: An, *Atriplex nummularia*; At, *Arabidopsis thaliana*; Ca, *Cicer arietinum*; Cp, *Cucurbita pepo*; Cs, *Cucumis sativus*; galga, *Ostreococcus lucimarinus*; Gm, *Glycine max*; Lj, *Lotus japonicus*; Mt, *Medicago truncatula*; Os, *Oryza sativa*; Pit, *Pinus taeda*; Pp, *Physcomitrella patens*; Pt, *Populus trichocarpa*; Sb, *Sorghum bicolor*; Vu, *Vigna unguiculata*; Vv, *Vitis vinifera*; Zm, *Zea mays*.

References cited in this table:

1. Bansal A, Sankararamakrishnan R: **Homology modeling of major intrinsic proteins in rice, maize and *Arabidopsis*: comparative analysis of transmembrane helix association and aromatic/arginine selectivity filters**. *BMC Struct Biol* 2007, **7**:27.
2. Ma JF, Tamai K, Yamaji N, Mitani N, Konishi S, Katsuhara M, Ishiguro M, Murata Y, Yano M: **A silicon transporter in rice**. *Nature* 2006, **440**:688-691.
3. Sakurai J, Ishikawa F, Yamaguchi T, Uemura M, Maeshima M: **Identification of 33 rice aquaporin genes and analysis of their expression and function**. *Plant Cell Physiol* 2005, **46**:1568-1577.
4. Mitani N, Yamaji N, Ma JF: **Characterization of substrate specificity of a rice silicon transporter, Lsi1**. *Pflugers Arch – Eur J Physiol* 2008, **456**:679-686.
5. Chaumont F, Barrieu F, Wojcik E, Chrispeels MJ, Jung R: **Aquaporins constitute a large and highly divergent protein family in maize**. *Plant Physiol* 2001, **125**:1206-1215.
6. Quigley F, Rosenberg JM, Shachar-Hill Y, Bohnert HJ: **From genome to function: the *Arabidopsis* aquaporins**. *Genome Biol* 2001, **3(1)**:research0001.1-0001.17.
7. Johanson U, Karlsson M, Johansson I, Gustavsson S., Sjövall S, Fraysse L, Weig AR, Kjellbom P: **The complete set of genes encoding major intrinsic proteins in *Arabidopsis* provides a framework for a new nomenclature for major intrinsic proteins in plants**. *Plant Physiol* 2001, **126**:1358-1369.
8. Bienert GP, Thorsen M, Schüssler MD, Nilsson HR, Wagner A, Tamás MJ, Jahn TP: **A subgroup of plant aquaporins facilitate the bi-directional diffusion of $As(OH)_3$ and $Sb(OH)_3$ across membranes**. *BMC Biol* 2008, **6**:26.
9. Kamiya T, Tanaka M, Mitani N, Ma JF, Maeshima M, Fujiwara T: ***NIP1;1*, an aquaporin homolog, determines the arsenite sensitivity of *Arabidopsis thaliana***. *J Biol Chem*

2009, **284**:2114-2120.

10. Fortin MG, Morrison NA, Verma DP: **Nodulin-26, a peribacteroid membrane nodulin is expressed independently of the development of the peribacteroid compartment**. *Nucleic Acids Res* 1987, **15**:813-824.

11. Choi WG, Roberts DM: *Arabidopsis* **NIP2;1, a major intrinsic protein transporter of lactic acid induced by anoxic stress**. *J Biol Chem* 2007, **282**:24209-24218.

12. Mizutani M, Watanabe S, Nakagawa T, Maeshima M: **Aquaporin NIP2;1 is mainly localized to the ER membrane and shows root-specific accumulation in** ***Arabidopsis thaliana***. *Plant Cell Physiol* 2006, **47**:1420-1426.

13. Ma JF, Yamaji N, Mitani N, Xu XY, Su YH, McGrath SP, Zhao FJ: **Transporters of arsenite in rice and their role in arsenic accumulation in rice grain**. *Proc Natl Acad Sci USA* 2008, **105**:9931-9935.

14. Isayenkov SV, Maathuis FJM: **The** ***Arabidopsis thaliana*** **aquaglyceroporin AtNIP7;1 is a pathway for arsenite uptake**. *FEBS Lett* 2008, **582**:1625-1628.

15. Danielson JÅH, Johanson U: **Unexpected complexity of the aquaporin gene family in the moss** ***Physcomitrella patens***. *BMC Plant Biol* 2008, **8**:45.

(B) The exon/intron lengths for each *NIP* gene in plants

NIPs	Exon1	Intron1	Exon2	Intron2	Exon3	Intron3	Exon4	Intron4	Exon5	Intron5	Exon6
OsNIP1;1	146	95	422	184	61	1364	222				
OsNIP1;2	159	931	259	1269	215	85	61	355	213		
OsNIP1;3	164	621	422	1709	61	1366	210				
OsNIP1;4	173	111	419	74	61	110	165				
**OsNIP2;1**	**149**	**143**	**224**	**1606**	**194**	**105**	**61**	**350**	**264**		
**OsNIP2;2**	**158**	**159**	**224**	**2094**	**194**	**113**	**61**	**713**	**255**		
OsNIP3;1	41	199	487	651	195						
OsNIP3;2	287	104	419	114	61	234	147				
OsNIP3;3	206	373	419	98	61	122	147				
OsNIP3;5	251	768	389	1303	61	102	147				
OsNIP4;1	173	106	686								
ZmNIP1;1	134	186	422	93	61	728	228				
ZmNIP2;1	143	156	224	1581	194	113	61	370	261		
ZmNIP2;2	158	134	224	2036	194	124	61	98	243		
ZmNIP2;3	167	120	224	1386	194	117	61	564	255		
ZmNIP3;1	224	1598	425	119	61	130	195				
AtNIP1;1	167	113	224	425	218	116	97	105	216		
AtNIP1;2	158	134	224	712	218	138	61	104	219		
AtNIP2;1	146	128	440	81	61	104	216				
AtNIP3;1	194	237	280	80	333						
AtNIP4;1	131	109	224	461	197	664	61	494	234		
AtNIP4;2	131	107	224	135	197	88	61	104	234		
AtNIP5;1	236	1726	419	265	61	78	195				
AtNIP6;1	242	97	224	207	194	78	61	80	192		
AtNIP7;1	140	177	224	237	183	211	72	101	201		
MtNIP2	116	94	230	461	199	357	62	291	208		
PpNIP3;1	231	147	225	151	195	437	62	92	193		

PpNIP5;1	228	207	195	232	61	147	202				
PpNIP5;2	255	227	195	244	61	147	202				
PpNIP5;3	246	195	195	140	61	154	190				
PpNIP6;1	222	205	192	133	61	122	145				
SbNIP1;1	146	521	422	120	61	940	231				
SbNIP1;2	239	108	203	133	61	134	213				
SbNIP1;3	155	455	422	1777	61	77	210				
SbNIP2;1	146	158	224	1753	194	105	61	458	264		
SbNIP2;2	155	277	224	2257	194	129	61	755	249		
SbNIP3;1	221	2865	425	108	61	75	195				
SbNIP3;2	263	95	419	91	61	111	147				
SbNIP3;3	434	146	61	122	144						
SbNIP3;5	683	93	61	94	147						
PtNIP2;1	102	253	225	1465	195	152	61	584	253		
PtNIP4;1	105	120	222	137	198	280	61	105	205		
PtNIP4;2	138	106	222	897	198	79	61	121	220		
PtNIP5;1	209	1877	420	83	61	1147	209				
PtNIP5;2	225	2102	420	245	61	555	196				
PtNIP6;1	225	117	224	90	195	628	61	664	205		
PtNIP6;2	225	122	225	89	195	1167	61	559	205		
PtNIP7;1	24	588	225	96	195	736	61	105	223		
GmNIP1;1	44	460	224	371	243	2356	3	164	213		
GmNIP1;3	116	462	224	376	197	1443	61	124	213		
GmNIP1;4	116	181	224	619	197	345	61	247	222		
GmNIP1;5	116	224	224	470	197	350	61	248	210		
GmNIP1;6	125	608	224	145	197	339	61	177	210		
GmNIP2;1	155	373	224	3068	194	208	61	225	249		
GmNIP2;2	143	2926	194	222	61	224	249				
GmNIP3;1	221	4252	431	74	61	76	195				

GmNIP4;1	218	1081	197	132	61	553	168				
GmNIP6;1	650	1452	61	1288	201						
GmNIP6;2	233	164	224	234	194	1519	61	1287	204		
VvNIP1;1	83	162	224	342	197	284	61	89	219		
VvNIP2;1	92	1020	194	83	61	82	255				
VvNIP4;1	53	29674	318	2501	100	180	61	92	135	3426	68
VvNIP5;1	218	719	419	160	61	663	195				
VvNIP6;1	239	163	425	533	61	511	336				
VvNIP7;1	188	106	224	94	194	92	61	94	210		
CsNIP1;1	126	103	225	308	198	95	62	181	220		
CsNIP2;1	147	263	225	2336	195	348	62	342	238		
CsNIP2;2	123	82	225	1741	195	97	62	192	181		
CsNIP3;1	336	80	62	140	196						
CsNIP4;2	117	469	222	1582	198	291	62	665	211		
CsNIP5;1	219	2812	420	78	62	1014	196				
CsNIP6;1	231	365	225	102	195	368	62	597	202		
CsNIP7;1	93	76	225	471	62	445	223				
galgaNIP	813										
