# Supplementary material for: Divergence in function and expression of the NOD26-like intrinsic proteins in plants
Source: BMC Genomics. 2009 Jul 15;10:313. doi: 10.1186/1471-2164-10-313 (PMC2726226; doi:10.1186/1471-2164-10-313)
Supplement: Additional file 2 — The multiple alignments of plant NIP protein sequences. These sequences were aligned using the program L-INS-i implemented in MAFFT v6.6. The alignments were shaded in the "Quantify Mode", and the residues were displayed in the "Difference Mode" with the "Diff/Consensus Line" style. Dots and "-" indicate similar residues and gaps on the alignment respectively. [file 1471-2164-10-313-S2.pdf]

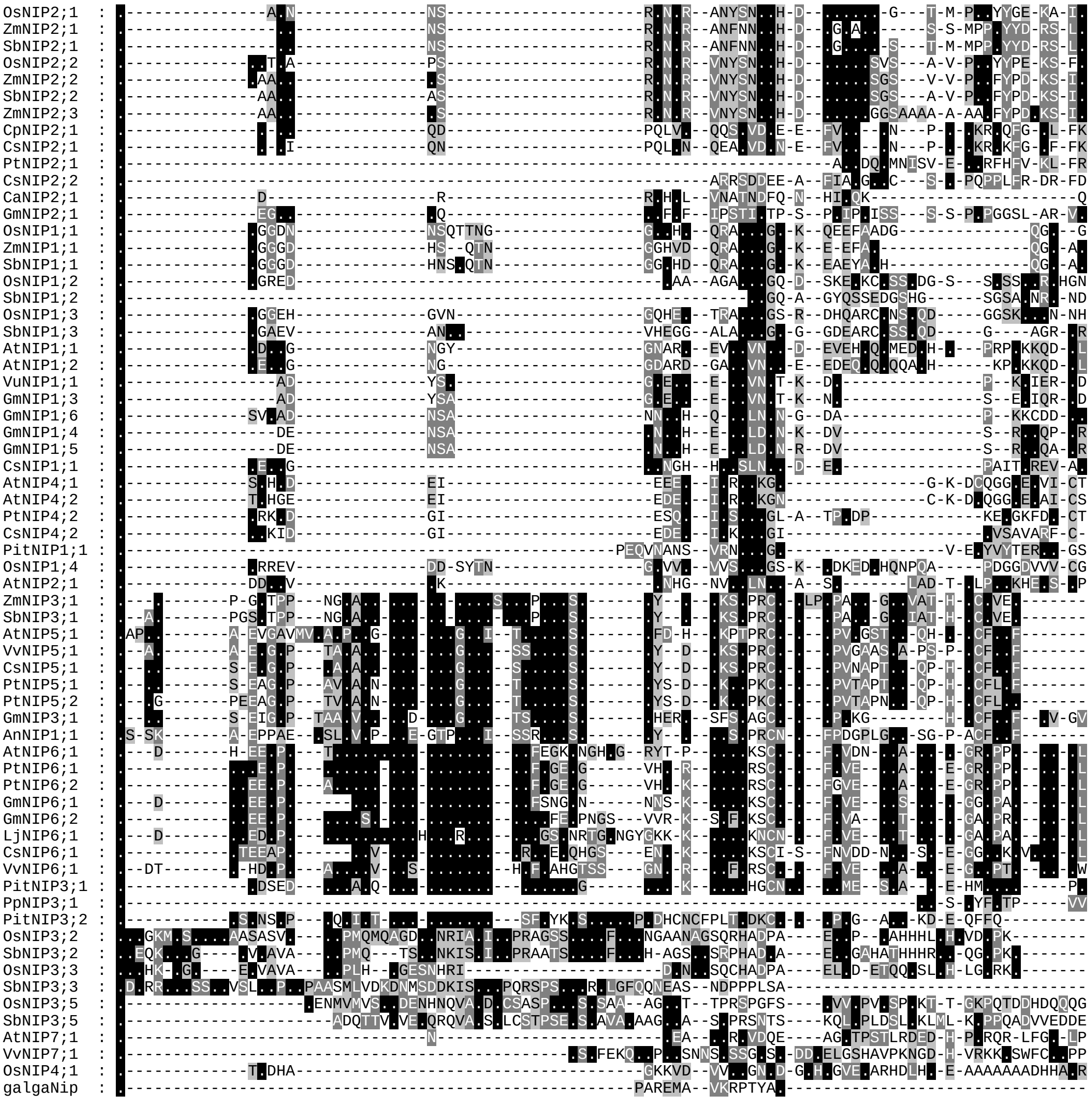
OsNIP2;1
ZmNIP2;1
SbNIP2;1
OsNIP2;2
ZmNIP2;2
SbNIP2;2
ZmNIP2;3
CpNIP2;1
CsNIP2;1
PtNIP2;1
CsNIP2;2
CaNIP2;1
GmNIP2;1
OsNIP1;1
ZmNIP1;1
SbNIP1;1
OsNIP1;2
SbNIP1;2
OsNIP1;3
SbNIP1;3
AtNIP1;1
AtNIP1;2
VuNIP1;1
GmNIP1;3
GmNIP1;6
GmNIP1;4
GmNIP1;5
CsNIP1;1
AtNIP4;1
AtNIP4;2
PtNIP4;2
CsNIP4;2
PitNIP1;1
OsNIP1;4
AtNIP2;1
ZmNIP3;1
SbNIP3;1
AtNIP5;1
VvNIP5;1
CsNIP5;1
PtNIP5;1
PtNIP5;2
GmNIP3;1
AnNIP1;1
AtNIP6;1
PtNIP6;1
PtNIP6;2
GmNIP6;1
GmNIP6;2
LjNIP6;1
CsNIP6;1
VvNIP6;1
PitNIP3;1
PpNIP3;1
PitNIP3;2
OsNIP3;2
SbNIP3;2
OsNIP3;3
SbNIP3;3
OsNIP3;5
SbNIP3;5
AtNIP7;1
VvNIP7;1
OsNIP4;1
galgaNip

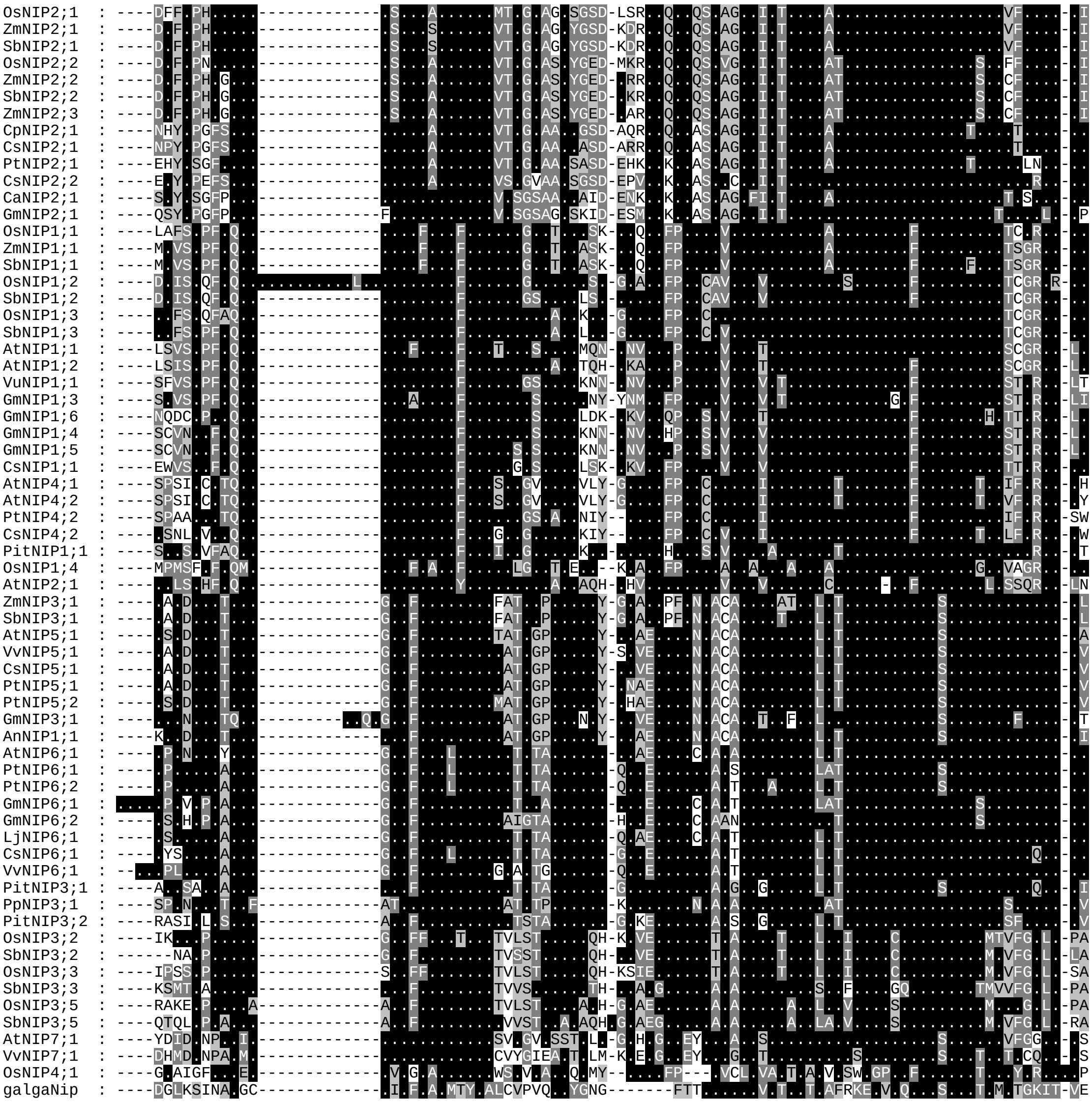
OsNIP2;1
ZmNIP2;1
SbNIP2;1
OsNIP2;2
ZmNIP2;2
SbNIP2;2
ZmNIP2;3
CpNIP2;1
CsNIP2;1
PtNIP2;1
CsNIP2;2
CaNIP2;1
GmNIP2;1
OsNIP1;1
ZmNIP1;1
SbNIP1;1
OsNIP1;2
SbNIP1;2
OsNIP1;3
SbNIP1;3
AtNIP1;1
AtNIP1;2
VuNIP1;1
GmNIP1;3
GmNIP1;6
GmNIP1;4
GmNIP1;5
CsNIP1;1
AtNIP4;1
AtNIP4;2
PtNIP4;2
CsNIP4;2
PitNIP1;1
OsNIP1;4
AtNIP2;1
ZmNIP3;1
SbNIP3;1
AtNIP5;1
VvNIP5;1
CsNIP5;1
PtNIP5;1
PtNIP5;2
GmNIP3;1
AnNIP1;1
AtNIP6;1
PtNIP6;1
PtNIP6;2
GmNIP6;1
GmNIP6;2
LjNIP6;1
CsNIP6;1
VvNIP6;1
PitNIP3;1
PpNIP3;1
PitNIP3;2
OsNIP3;2
SbNIP3;2
OsNIP3;3
SbNIP3;3
OsNIP3;5
SbNIP3;5
AtNIP7;1
VvNIP7;1
OsNIP4;1
galgaNip

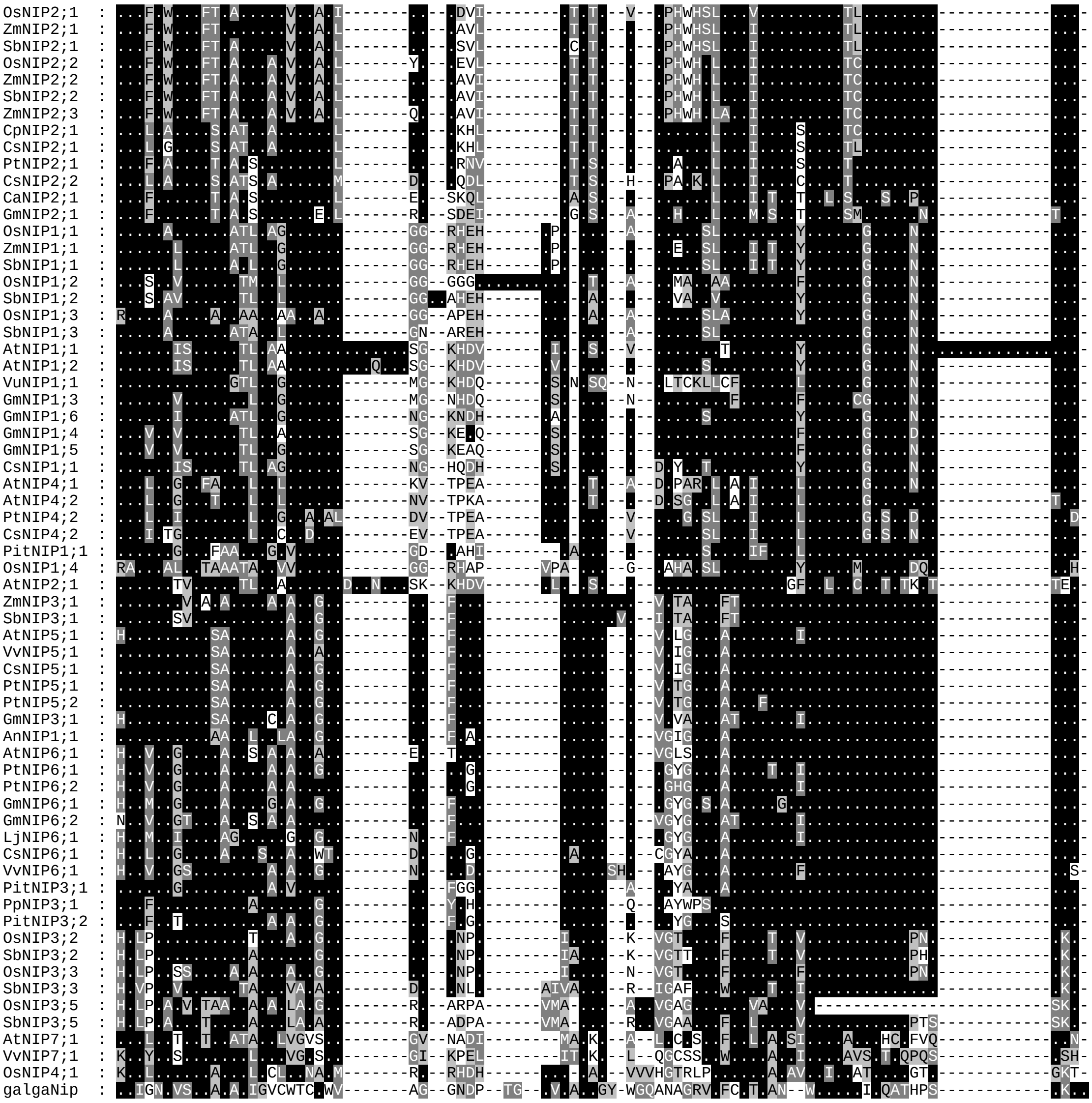
OsNIP2;1
ZmNIP2;1
SbNIP2;1
OsNIP2;2
ZmNIP2;2
SbNIP2;2
ZmNIP2;3
CpNIP2;1
CsNIP2;1
PtNIP2;1
CsNIP2;2
CaNIP2;1
GmNIP2;1
OsNIP1;1
ZmNIP1;1
SbNIP1;1
OsNIP1;2
SbNIP1;2
OsNIP1;3
SbNIP1;3
AtNIP1;1
AtNIP1;2
VuNIP1;1
GmNIP1;3
GmNIP1;6
GmNIP1;4
GmNIP1;5
CsNIP1;1
AtNIP4;1
AtNIP4;2
PtNIP4;2
CsNIP4;2
PitNIP1;1
OsNIP1;4
AtNIP2;1
ZmNIP3;1
SbNIP3;1
AtNIP5;1
VvNIP5;1
CsNIP5;1
PtNIP5;1
PtNIP5;2
GmNIP3;1
AnNIP1;1
AtNIP6;1
PtNIP6;1
PtNIP6;2
GmNIP6;1
GmNIP6;2
LjNIP6;1
CsNIP6;1
VvNIP6;1
PitNIP3;1
PpNIP3;1
PitNIP3;2
OsNIP3;2
SbNIP3;2
OsNIP3;3
SbNIP3;3
OsNIP3;5
SbNIP3;5
AtNIP7;1
VvNIP7;1
OsNIP4;1
galgaNip

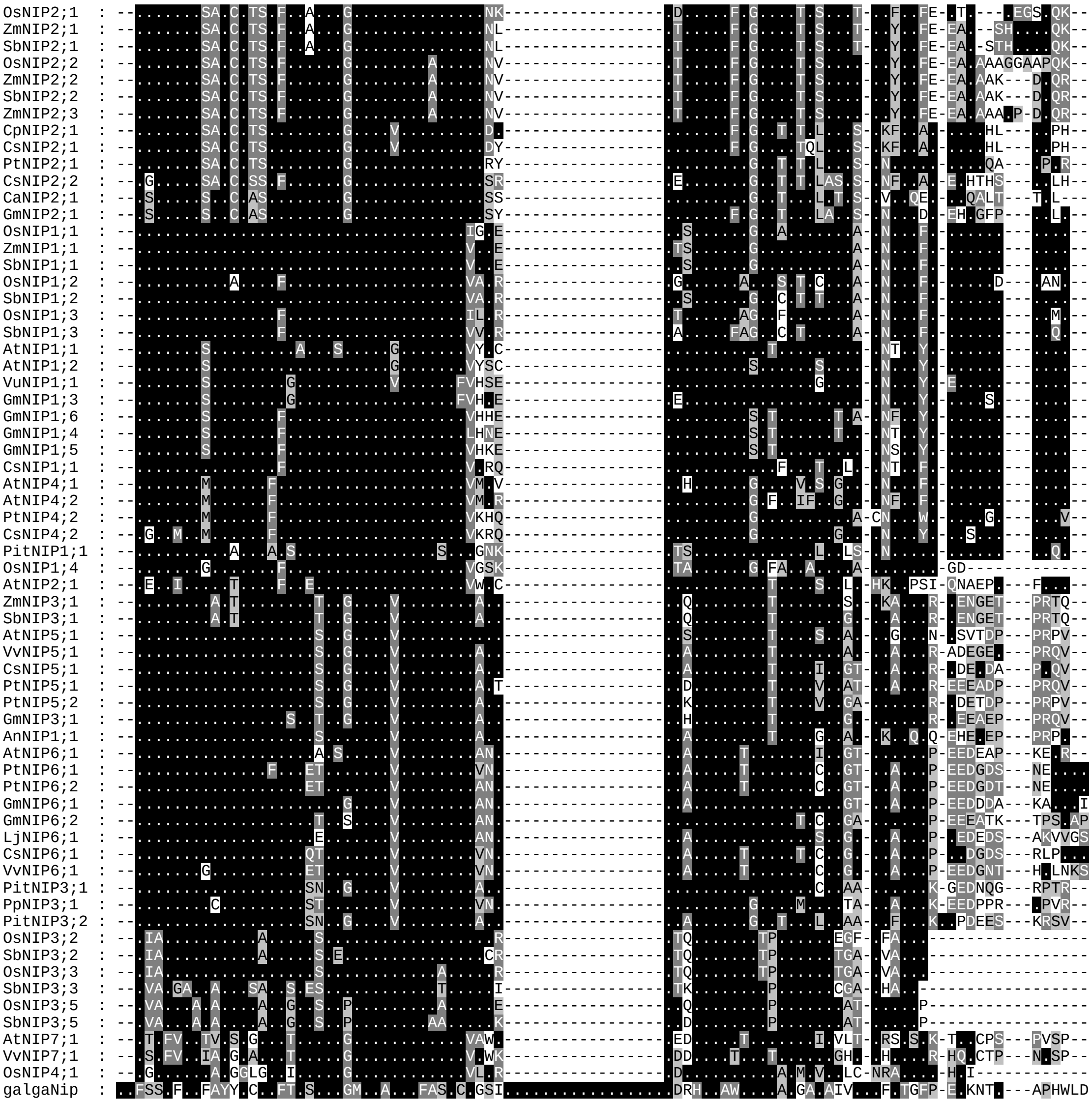
OsNIP2;1
ZmNIP2;1
SbNIP2;1
OsNIP2;2
ZmNIP2;2
SbNIP2;2
ZmNIP2;3
CpNIP2;1
CsNIP2;1
PtNIP2;1
CsNIP2;2
CaNIP2;1
GmNIP2;1
OsNIP1;1
ZmNIP1;1
SbNIP1;1
OsNIP1;2
SbNIP1;2
OsNIP1;3
SbNIP1;3
AtNIP1;1
AtNIP1;2
VuNIP1;1
GmNIP1;3
GmNIP1;6
GmNIP1;4
GmNIP1;5
CsNIP1;1
AtNIP4;1
AtNIP4;2
PtNIP4;2
CsNIP4;2
PitNIP1;1
OsNIP1;4
AtNIP2;1
ZmNIP3;1
SbNIP3;1
AtNIP5;1
VvNIP5;1
CsNIP5;1
PtNIP5;1
PtNIP5;2
GmNIP3;1
AnNIP1;1
AtNIP6;1
PtNIP6;1
PtNIP6;2
GmNIP6;1
GmNIP6;2
LjNIP6;1
CsNIP6;1
VvNIP6;1
PitNIP3;1
PpNIP3;1
PitNIP3;2
OsNIP3;2
SbNIP3;2
OsNIP3;3
SbNIP3;3
OsNIP3;5
SbNIP3;5
AtNIP7;1
VvNIP7;1
OsNIP4;1
galgaNip

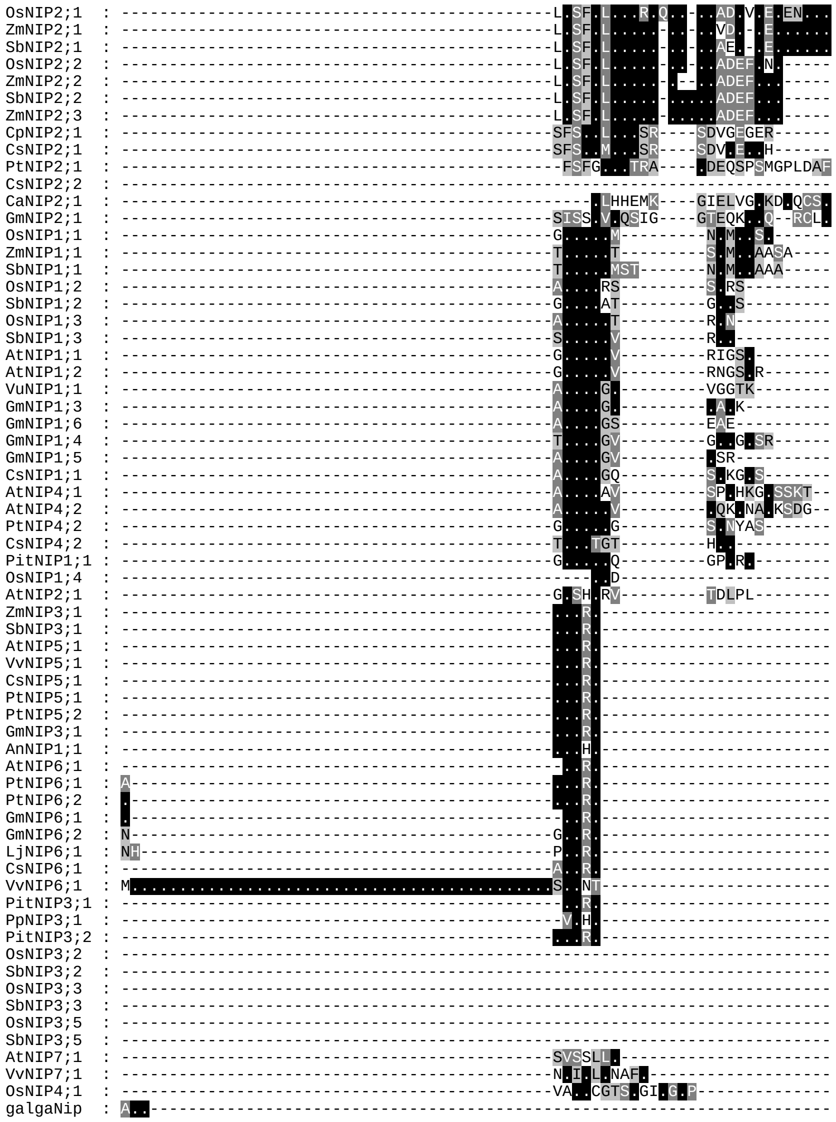
OsNIP2;1
ZmNIP2;1
SbNIP2;1
OsNIP2;2
ZmNIP2;2
SbNIP2;2
ZmNIP2;3
CpNIP2;1
CsNIP2;1
PtNIP2;1
CsNIP2;2
CaNIP2;1
GmNIP2;1
OsNIP1;1
ZmNIP1;1
SbNIP1;1
OsNIP1;2
SbNIP1;2
OsNIP1;3
SbNIP1;3
AtNIP1;1
AtNIP1;2
VuNIP1;1
GmNIP1;3
GmNIP1;6
GmNIP1;4
GmNIP1;5
CsNIP1;1
AtNIP4;1
AtNIP4;2
PtNIP4;2
CsNIP4;2
PitNIP1;1
OsNIP1;4
AtNIP2;1
ZmNIP3;1
SbNIP3;1
AtNIP5;1
VvNIP5;1
CsNIP5;1
PtNIP5;1
PtNIP5;2
GmNIP3;1
AnNIP1;1
AtNIP6;1
PtNIP6;1
PtNIP6;2
GmNIP6;1
GmNIP6;2
LjNIP6;1
CsNIP6;1
VvNIP6;1
PitNIP3;1
PpNIP3;1
PitNIP3;2
OsNIP3;2
SbNIP3;2
OsNIP3;3
SbNIP3;3
OsNIP3;5
SbNIP3;5
AtNIP7;1
VvNIP7;1
OsNIP4;1
galgaNip

Additional file 2 The multiple alignments of plant NIP protein sequences. These sequences were aligned using the program L-INS-i implemented in MAFFT v6.6. The alignments were shaded in the “Quantify Mode”, and the residues were displayed in the “Difference Mode” with the “Diff / Consensus Line” style. Dots and “-“ indicate similar residues and gaps on the alignment respectively.
