# Supplementary material for: Divergence in function and expression of the NOD26-like intrinsic proteins in plants
Source: BMC Genomics. 2009 Jul 15;10:313. doi: 10.1186/1471-2164-10-313 (PMC2726226; doi:10.1186/1471-2164-10-313)
Supplement: Additional file 3 — Phylogenetic tree reconstructed using plant NIP nucleotide sequences. The number beside the branches represents bootstrap values ≥ 300 based on 500 resamplings. The scale bar shows total nucleotide distance. The NIP homologue in the green alga Ostreococcus lucimarinus (defined as galgaNIP) is used as outgroup sequence to root the tree. Branches with rates of numbers of nonsynonymous and synonymous substitutions >1, are indicated by red thick lines. [file 1471-2164-10-313-S3.doc]

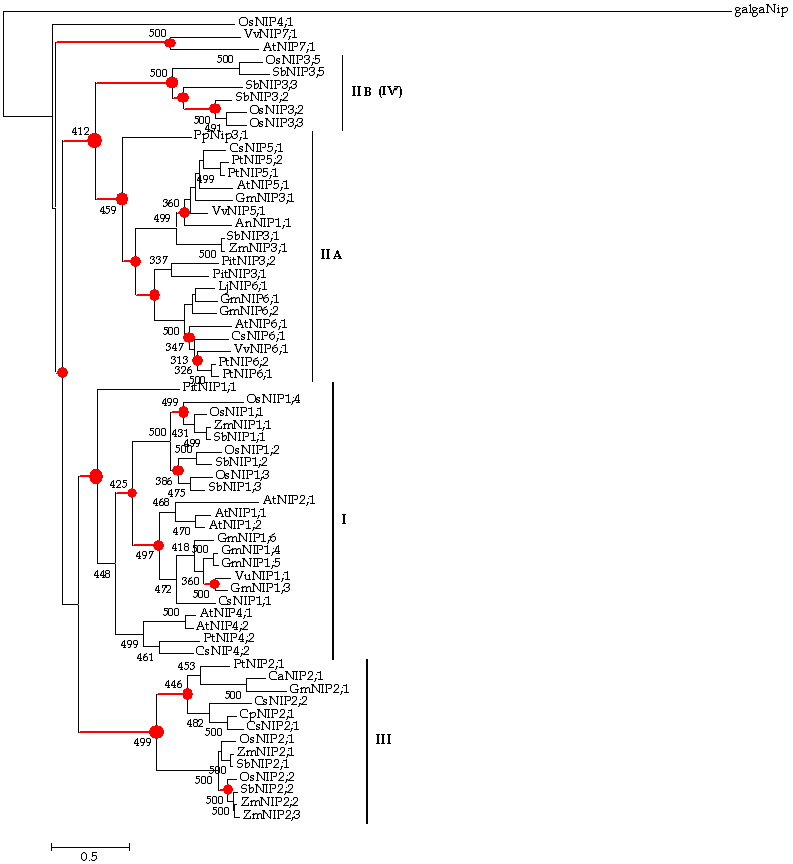


Additional file 3

**Phylogenetic tree reconstructed using plant *NIP* nucleotide sequences**. The *number* beside the branches represents bootstrap values ≥300 based on 500 resamplings. The scale bar shows total nucleotide distance. The *NIP* homolog in the green alga *Ostreococcus lucimarinus* (defined as *galgaNIP*) is used as outgroup sequence to root the tree. Branches with rates of numbers of nonsynonymous and synonymous substitutions >1, are indicated by red thick lines. To identify the species of origin for each *NIP* gene, a species acronym is included before the gene name: An, *Atriplex nummularia*; At, *Arabidopsis thaliana*; Ca, *Cicer arietinum*; Cp, *Cucurbita pepo*; Cs, *Cucumis sativus*; galga, *Ostreococcus lucimarinus*; Gm, *Glycine max*; Lj, *Lotus japonicus*; Os, *Oryza sativa*; Pit, *Pinus taeda*; Pp, *Physcomitrella patens*; Pt, *Populus trichocarpa*; Sb, *Sorghum bicolor*; Vu, *Vigna unguiculata*; Vv, *Vitis vinifera*; Zm, *Zea mays*.
