# Supplementary material for: Divergence in function and expression of the NOD26-like intrinsic proteins in plants
Source: BMC Genomics. 2009 Jul 15;10:313. doi: 10.1186/1471-2164-10-313 (PMC2726226; doi:10.1186/1471-2164-10-313)
Supplement: Additional file 5 — The expression patterns of Arabidopsis AtNIPs during the plant life cycle. [file 1471-2164-10-313-S5.doc]

Additional file 5

The expression patterns of *Arabidopsis* *AtNIPs* during the plant life cycle.
