# Supplementary material for: Divergence in function and expression of the NOD26-like intrinsic proteins in plants
Source: BMC Genomics. 2009 Jul 15;10:313. doi: 10.1186/1471-2164-10-313 (PMC2726226; doi:10.1186/1471-2164-10-313)
Supplement: Additional file 6 — The diversity of expression profiles of Arabidopsis AtNIP genes in root cell-types after treatment with salt. [file 1471-2164-10-313-S6.doc]

Additional file 6

The diversity of expression profiles of *Arabidopsis AtNIP* genes in root cell-types after treatment with salt.
