# Supplementary material for: Divergence in function and expression of the NOD26-like intrinsic proteins in plants
Source: BMC Genomics. 2009 Jul 15;10:313. doi: 10.1186/1471-2164-10-313 (PMC2726226; doi:10.1186/1471-2164-10-313)

Additional file 7

(A) Analysis of *cis*-acting elements in the 1000bp sequence upstream of the translation initiation codon in plant *NIP* genes

| Element | Function | O. sativa | | | | | | | | | | | V. vinifera | | | | | |
| --- | --- | --- | --- | --- | --- | --- | --- | --- | --- | --- | --- | --- | --- | --- | --- | --- | --- | --- |
|  |  | 1;1 | *1;2* | *1;3* | *1;4* | *2;1* | *2;2* | *3;1* | *3;2* | *3;3* | 3;5 | *4;1* | *1;1* | *2;1* | *4;1* | *5;1* | *6;1* | *7;1* |
| 5′-UTR Py-rich stretch ^[14]^ | Conferring high transcription levels | 3 |  | 2 |  |  |  |  |  |  | 1 |  |  | 2 |  | 2 | 1 |  |
| ACE ^[15]^ | Light | 1 |  | 1 |  |  |  |  |  |  |  |  | 1 |  |  |  |  |  |
| AE-box ^[19]^ | Light |  |  | 1 | 1 | 1 |  | 1 |  |  | 1 |  |  | 1 |  |  |  | 1 |
| G-Box ^[10]^ | Light | 2 | 2 | 1 | 1 | 2 | 7 | 2 | 1 |  | 1 |  | 1 | 2 |  | 1 |  | 1 |
| GAG-motif ^[11]^ | Light | 1 | 1 | 1 |  | 1 |  | 2 | 1 | 2 |  | 2 |  | 2 |  |  | 1 | 2 |
| MNF1 | Light |  |  | 1 |  |  |  |  |  |  |  |  |  |  |  |  |  |  |
| AT1-motif | Light | 1 |  |  |  |  |  |  |  |  |  |  |  |  |  |  | 1 |  |
| GATA-motif ^[1]^ | Light | 1 |  |  |  |  |  | 1 |  |  |  |  | 1 |  |  |  |  | 1 |
| I-box ^[4]^ | Light | 1 |  |  | 1 | 1 |  |  |  | 2 |  | 1 |  | 1 |  |  | 2 | 1 |
| L-box | Light | 1 |  |  |  |  |  |  |  | 1 |  |  |  |  |  |  |  |  |
| Chs-CMA2a | Light | 1 |  |  |  |  |  |  |  |  |  |  | 1 |  |  |  |  |  |
| TCCC-motif ^[20]^ | Light |  | 1 |  |  | 1 | 1 |  |  |  |  | 1 |  |  |  |  |  |  |
| RbcS-CMA7a | Light |  | 1 |  |  |  |  | 1 |  |  |  |  |  |  |  |  |  |  |
| Box I ^[23]^ | Light |  |  |  | 1 |  |  | 1 | 1 | 3 |  |  |  |  |  | 1 | 2 |  |
| GT1-motif ^[2]^ | Light |  |  |  | 2 |  |  |  | 1 | 1 | 1 | 2 | 1 | 1 |  |  |  |  |
| Box II | Light |  |  |  |  | 1 |  |  |  |  |  |  |  |  |  |  |  |  |
| Chs-Unit 1 m1 | Light |  |  |  |  |  | 1 |  |  | 1 |  |  |  |  |  |  |  |  |
| AAAC-motif | Light |  |  |  |  |  |  | 1 |  |  |  |  |  |  |  |  |  |  |
| GTGGC-motif | Light |  |  |  |  |  |  | 1 |  |  |  |  |  |  |  |  |  |  |
| ATCT-motif | Light |  |  |  |  |  |  |  | 1 |  | 1 |  |  |  |  |  |  | 1 |
| GA-box | Light |  |  |  |  |  |  |  |  | 1 |  |  |  |  |  |  |  |  |
| GA-motif ^[26]^ | Light |  |  |  |  |  |  |  |  |  | 1 |  |  | 1 | 1 | 1 |  |  |
| LAMP-element | Light |  |  |  |  |  |  |  |  |  |  |  |  | 1 |  |  |  |  |
| Box 4 ^[12]^ | Light | 6 |  |  |  | 2 |  | 3 | 7 |  | 4 | 1 | 1 | 2 | 1 |  | 2 | 1 |
| MRE | Light |  |  |  |  |  |  |  |  |  |  | 1 |  |  |  |  |  |  |
| TGG-motif | Light |  |  |  |  |  |  |  |  |  |  |  |  |  |  |  |  | 1 |
| Gap-box | Light |  |  |  |  |  |  |  |  |  |  |  |  |  |  | 1 |  |  |
| 3-AF1 binding site | Light |  |  |  |  |  |  |  |  |  |  | 1 | 1 |  |  | 1 | 1 |  |
| ABRE ^[6]^ | ABA | 1 | 3 |  | 1 |  | 3 |  |  |  | 1 |  |  |  |  |  |  |  |
| CE3 | ABA |  |  |  |  |  | 1 |  |  |  |  |  |  |  |  |  |  |  |
| TGA-element ^[21]^ | Auxin |  |  |  | 1 | 1 |  | 1 |  |  |  |  |  | 1 |  |  |  |  |
| TCA-element ^[24]^ | Salicylic acid |  |  |  |  |  |  | 1 |  |  |  |  | 1 | 2 | 1 | 1 | 1 |  |
| GARE-motif ^[3]^ | Gibberellin | 2 | 2 |  |  | 1 |  |  |  |  |  | 1 |  | 3 |  | 2 |  |  |
| P-box ^[22]^ | Gibberellin |  |  |  |  | 2 |  |  |  |  |  | 2 |  |  |  | 2 | 1 |  |
| ARE ^[13]^ | Anaerobic induction | 1 | 1 | 1 | 1 |  | 2 | 2 | 4 | 2 |  | 1 | 2 | 1 | 2 | 1 |  |  |
| GC-motif ^[13]^ | Anoxic inducibility |  |  |  |  | 1 | 2 |  |  |  |  |  | 1 |  |  |  |  |  |
| Box-W1 ^[16]^ | Fungal elicitor responsive element | 1 |  | 1 |  |  |  |  | 2 |  |  | 1 | 1 | 2 | 1 |  | 1 | 1 |
| HSE ^[24]^ | Heat |  |  |  |  |  |  |  |  |  | 1 | 1 | 1 | 1 |  |  |  |  |
| LTR ^[7]^ | Low-temperature |  |  |  |  | 1 |  |  | 1 |  |  |  |  |  |  |  |  |  |
| MBS ^[5]^ | Drought | 4 |  | 2 |  | 3 | 1 | 2 | 3 |  |  | 4 |  | 2 | 1 |  | 1 |  |
| TC-rich repeats ^[17]^ | Defense and stress | 2 |  |  | 1 |  |  |  |  |  | 1 |  | 1 |  | 2 | 2 |  |  |
| WUN-motif | Wound-responsive element |  |  |  | 1 |  |  |  |  |  |  |  |  |  |  |  |  |  |
| GCN4-motif ^[25]^ | Endosperm |  | 1 | 2 |  |  |  | 1 |  |  | 2 |  |  |  |  |  |  | 1 |
| RY-element | Seed-specific regulation |  |  | 1 |  |  |  |  | 1 |  |  |  |  |  |  |  |  |  |
| CAT-box | Meristem expression | 1 |  |  |  |  |  |  |  | 1 |  |  |  |  | 1 |  | 1 |  |
| dOCT | meristem specific activation |  |  |  |  | 1 |  |  |  |  |  |  |  |  |  |  |  |  |
| O2-site ^[18]^ | Zein metabolism regulation | 1 |  |  |  |  |  | 1 | 1 | 3 |  |  | 1 | 1 | 1 |  | 1 | 1 |
| MSA-like | Cell cycle |  |  |  |  |  |  |  |  | 1 |  |  |  |  |  |  |  | 1 |
| As-2-box ^[29]^ | Shoot-specific expression |  |  | 1 |  |  |  |  |  |  |  | 1 |  | 2 |  |  |  |  |
| AC-I ^[8]^ | Xylem expression | 2 |  |  |  |  |  |  |  |  |  |  | 1 |  | 2 |  |  |  |
| AC-II ^[8]^ | Xylem expression |  |  |  |  |  |  |  |  |  |  |  | 1 |  |  |  | 1 |  |
| A-box | Conserved in alpha-amylase promoters |  |  |  | 1 |  |  |  |  |  |  |  |  |  |  |  |  |  |
| HD-Zip1 | Differentiation of the palisade mesophyll cells |  |  |  |  |  |  |  |  |  |  |  |  |  | 1 |  |  |  |
| HD-Zip2 | Control of leaf morphology development |  |  |  |  |  |  |  |  |  |  |  |  |  | 1 |  |  |  |
| Circadian ^[9]^ | Circadian control | 1 | 1 |  | 1 | 1 |  | 1 | 2 |  |  |  |  |  | 1 |  |  | 1 |

Additional file 7

Continued.

| Element | Function | S. bicolor | | | | | | | | | P. trichocarpa | | |  |  |  | |  |
| --- | --- | --- | --- | --- | --- | --- | --- | --- | --- | --- | --- | --- | --- | --- | --- | --- | --- | --- |
|  |  | 1;1 | *1;2* | *1;3* | *2;1* | *2;2* | *3;1* | *3;2* | *3;3* | 3;5 | *2;1* | *4;1* | *4;2* | *5;1* | *5;2* | *6;1* | *6;2* | *7;1* |
| 5′-UTR Py-rich stretch ^[14]^ | Conferring high transcription levels | 1 |  |  |  |  |  |  |  |  |  |  | 1 | 1 | 2 |  | 3 | 1 |
| ACE ^[15]^ | Light |  | 1 | 1 |  |  |  |  |  |  |  |  |  |  |  |  |  |  |
| AE-box ^[19]^ | Light |  |  | 1 |  |  |  |  | 1 |  |  |  |  | 1 |  | 1 |  | 1 |
| G-Box ^[10]^ | Light | 2 | 8 |  | 6 |  |  | 3 | 2 |  |  |  | 2 |  | 1 |  | 2 |  |
| GAG-motif ^[11]^ | Light |  | 3 | 1 |  |  | 2 | 2 |  | 1 |  |  | 1 | 1 | 1 | 2 | 2 | 2 |
| MNF1 | Light |  | 1 |  |  |  |  |  |  | 1 |  |  |  |  |  |  |  |  |
| AT1-motif | Light |  |  |  |  |  |  |  |  |  |  |  |  | 2 |  |  |  |  |
| GATA-motif ^[1]^ | Light | 1 | 1 |  |  | 1 | 1 |  |  | 1 |  |  |  |  | 1 |  |  |  |
| I-box ^[4]^ | Light |  |  |  |  | 1 | 2 | 1 |  | 1 |  |  |  |  |  |  |  |  |
| L-box | Light |  | 1 |  |  |  |  |  |  |  |  |  |  |  |  |  |  |  |
| Chs-CMA2a | Light |  |  |  |  |  |  |  |  |  |  |  |  |  |  | 1 | 1 |  |
| TCCC-motif ^[20]^ | Light | 1 | 2 |  | 2 | 1 | 1 |  | 2 |  |  |  |  |  |  |  |  |  |
| Box I ^[23]^ | Light | 1 |  |  |  | 1 |  | 1 | 1 |  |  |  | 4 | 1 |  | 1 |  |  |
| GT1-motif ^[2]^ | Light | 2 |  |  | 2 | 1 |  |  |  |  |  | 1 | 1 |  |  |  |  | 1 |
| AAAC-motif | Light |  |  |  |  |  | 1 |  |  |  |  |  |  |  |  |  |  |  |
| GTGGC-motif | Light |  |  |  |  |  |  |  |  |  |  |  |  |  | 1 |  |  |  |
| ATCT-motif | Light |  |  |  |  |  |  |  |  |  |  |  |  |  | 1 |  |  |  |
| GA-motif ^[26]^ | Light |  |  |  |  |  | 1 | 1 |  | 2 |  |  |  | 1 | 1 |  |  |  |
| LAMP-element | Light |  |  |  |  |  |  |  |  |  |  |  |  |  |  |  | 1 |  |
| TGG-motif | Light |  | 1 |  |  |  |  |  |  |  |  |  |  |  |  | 1 |  |  |
| Gap-box | Light |  |  |  |  |  |  |  |  |  |  |  |  |  |  |  | 1 |  |
| Box 4 ^[12]^ | Light |  |  |  |  |  |  |  | 1 | 1 | 1 | 1 | 3 | 1 | 1 | 3 | 3 |  |
| TCT-motif ^[27]^ | Light | 1 |  |  |  |  |  |  |  |  | 1 | 1 | 1 | 1 | 1 |  |  | 1 |
| CAG-motig | Light |  |  |  | 1 |  |  |  |  |  |  |  |  |  |  |  |  |  |
| 3-AF1 binding site | Light |  |  |  |  |  |  |  |  |  |  |  | 1 |  |  |  |  |  |
| ABRE ^[6]^ | ABA |  | 2 |  | 3 |  |  | 2 |  |  |  |  |  |  |  |  |  |  |
| TCA-element ^[24]^ | Salicylic acid |  | 2 |  |  | 1 |  |  | 2 |  | 2 | 2 | 3 | 1 | 2 |  | 2 |  |
| TGA-element ^[21]^ | Auxin |  | 1 |  |  | 1 |  |  |  | 1 | 1 | 1 |  |  |  |  |  |  |
| AuxRR-core | Auxin |  |  |  |  |  |  |  |  |  |  | 1 |  |  |  |  |  |  |
| GARE-motif ^[3]^ | Gibberellin | 1 | 1 |  |  |  |  | 3 |  |  |  |  |  | 1 |  |  |  |  |
| TATC-box | Gibberellin |  |  |  |  |  |  |  |  |  |  |  |  |  |  |  | 1 |  |
| P-box ^[22]^ | Gibberellin |  |  |  |  |  |  |  |  |  |  | 1 | 1 | 1 |  |  | 1 | 1 |
| ERE ^[28]^ | Ethylene-responsive element |  |  |  |  | 1 |  |  | 1 |  |  |  | 2 |  |  |  |  |  |
| HSE ^[24]^ | Heat | 3 |  |  |  | 1 |  |  |  |  | 1 | 1 |  | 3 | 1 |  | 1 |  |
| ARE ^[13]^ | Anaerobic induction |  | 1 |  |  | 1 | 3 | 2 | 1 |  | 2 | 2 | 1 | 2 | 1 | 1 | 1 |  |
| GC-motif ^[13]^ | Anoxic specific inducibility |  | 2 |  | 1 | 1 |  |  |  |  |  |  |  |  |  |  |  | 2 |
| LTR ^[7]^ | Low-temperature | 1 |  |  |  |  |  |  | 1 |  |  |  | 1 |  |  |  |  |  |
| Box-W1 ^[16]^ | Fungal elicitor responsive element |  | 2 |  |  |  | 1 |  |  |  |  | 1 |  |  |  |  |  | 1 |
| MBS ^[5]^ | Drought | 1 | 4 | 2 | 3 | 1 | 3 | 1 | 4 | 1 | 1 | 1 |  | 1 | 1 |  | 3 | 2 |
| TC-rich repeats ^[17]^ | Defense and stress |  |  | 1 |  | 3 |  |  | 1 | 1 | 1 | 1 | 2 |  | 2 | 2 | 1 |  |
| WUN-motif | Wound-responsive element |  |  | 1 |  |  |  |  |  |  |  |  |  | 1 |  |  |  |  |
| GCN4-motif ^[25]^ | Endosperm |  |  |  |  |  |  |  | 1 | 1 |  |  |  |  | 1 | 1 |  |  |
| CAT-box | Meristem expression |  | 1 |  |  |  |  |  | 1 | 2 |  | 3 |  |  |  |  |  | 1 |
| CCGTCC-box | Meristem specific activation | 1 |  |  | 2 | 3 |  |  | 1 |  |  |  |  |  |  | 1 | 1 |  |
| O2-site ^[18]^ | Zein metabolism regulation |  | 1 | 1 |  |  | 1 | 2 | 3 |  |  |  | 1 | 1 | 1 |  |  |  |
| As-2-box ^[29]^ | Shoot-specific expression |  |  | 1 |  |  | 1 | 1 | 1 |  |  |  |  | 1 | 1 |  |  |  |
| AC-I ^[8]^ | Xylem expression |  |  | 1 |  |  | 1 |  |  |  |  |  |  |  |  |  |  |  |
| AC-II ^[8]^ | Xylem expression |  |  | 1 |  |  |  |  |  | 1 |  |  |  |  |  |  |  |  |
| HD-Zip1 | Differentiation of the palisade mesophyll cells |  |  |  |  |  |  | 1 |  |  |  |  |  |  |  |  | 1 |  |
| HD-Zip2 | Control of leaf morphology development |  |  |  |  |  |  | 1 |  |  |  |  |  |  |  |  | 1 |  |
| Circadian ^[9]^ | Circadian control | 1 | 1 | 1 |  |  | 3 |  | 2 | 1 |  |  | 1 |  |  | 1 |  |  |

References cited in this table:

1. Gilmartin PM, Sarokin L, Memelink J, Chua NH: **Molecular light switches for plant genes**. *Plant Cell* 1990, **2**:369-378.
2. Villain P, Mache R, Zhou DX: **The mechanism of GT element-mediated cell type-specific transcriptional control**. *J Biol Chem* 1996, **271**:32593-32598.
3. Ogawa M, Hanada A, Yamauchi Y, Kuwahara A, Kamiya Y, Yamaguchi S: **Gibberellin biosynthesis and response during *Arabidopsis* seed germination**. *Plant Cell* 2003, **15**:1591-1604.
4. Terzaghi WB, Cashmore AR: **Light-regulated transcription**. *Annu Rev Plant Physiol Plant Mol Biol* 1995, **46**:445-474.
5. Yamaguchi-Shinozaki K, Shinozaki K: ***Arabidopsis* DNA encoding two desiccation-responsive rd29 genes**. *Plant Physiol* 1993, **101**:1119-1120.
6. Simpson SD, Nakashima K, Narusaka Y, Seki M, Shinozaki K, Yamaguchi-Shinozaki K: **Two different novel *cis*-acting elements of erd1, a clpA homologous *Arabidopsis* gene function in induction by dehydration stress and dark-induced senescence**. *Plant J* 2003, **33**:259-270.
7. Baker SS, Wilhelm KS, Thomashow MF: **The 5’-region of *Arabidopsis thaliana* core15a has *cis*-acting elements that confer cold-, drought- and ABA-regulated gene expression**. *Plant Mol Biol* 1994, **24**:701-713.
8. Hatton D, Sablowski R, Yung MH, Smith C, Schuch W, Bevan M: **Two classes of *cis* sequences contribute to tissue-specific expression of a PAL2 promoter in transgenic tobacco**. *Plant J* 1995, **7**:859-876.
9. Pichersky E, Bernatzky R, Tanksley SD, Breidenbach RB, Kausch AP, Cashmore AR: **Molecular characterization and genetic mapping of two clusters of genes encoding chlorophyll a/b-biding proteins in *Lycopersicon esculentum* (tomato)**. *Gene* 1985, **40**:247-258.
10. Sommer H, Saedler H: **Structure of the chalcone synthase gene of *Antirrhinum majus***. *Mol Gen Genet* 1986, **202**:429-434.
11. Werneke JM, Chatfield JM, Ogren WL: **Alternative mRNA splicing generates the two ribulosebisphosphate carboxylase/oxygenase activase polypeptides in spinach and *Arabidopsis***. *Plant Cell* 1989, **1**:815-825.
12. Lois R, Dietrich A, Hahlbrock K, Schulz W: **A phenylalanine ammonia-lyase gene from parsley: structure, regulation and identification of elicitor and light responsive *cis*-acting elements**. *EMBO J* 1989, **8**:1641-1648.
13. Manjunath S, Sachs MM: **Molecular characterization and promoter analysis of the maize cytosolic glyceraldehyde 3-phosphate dehydrogenase gene family and its expression during anoxia**. *Plant Mol Biol* 1997, **33**:97-112.
14. Daraselia ND, Tarchevskaya S, Narita JO: **The promoter for tomato 3-hydroxy-3-methylglutaryl coenzyme A reductase gene 2 has unusual regulatory elements that direct high-level expression**. *Plant Physiol* 1996, **112**:727-733.
15. Feldbrugge M, Sprenger M, Dinkelbach M, Yazaki K, Harter K, Weisshaar B: **Functional analysis of a light-responsive plant bZIP transcriptional regulator**. *Plant Cell* 1994, **6**:1607-1621.
16. Rushton PJ, Torres JT, Parniske M, Wernert P, Hahlbrock K, Somssich I.E: **Interaction of elicitor-induced DNA-binding proteins with elicitor response elements in the promoters of parsley PR1 genes**. *EMBO J* 1996, **15**:5690-5700.
17. Diaz-De-Leon F, Klotz KL, Lagrimini M: **Nucleotide sequence of the tobacco (Nicotiana tabacum) anionic peroxidase gene**. *Plant Physiol* 1993, **101**:1117-1118.
18. Hartings H, Lazzaroni N, Marsan PA, Aragay A, Thompson R, Salamini F, Di Fonzo N, Palau J, Motto M: **The b-32 protein from maize endosperm: characterization of genomic sequences encoding two alternative central domains**. *Plant Mol Biol* 1990, **14**:1031-1040.
19. Conley TR, Park SC, Kwon HB, Peng HS, Shih MC: **Characterization of *cis*-acting elements in light regulation of the nuclear gene encoding the A subunit of chloroplast isozymes of glyceraldehyde-3-phosphate dehydrogenase from *Arabidopsis thaliana***. *Mol Cell Biol* 1994, **14**:2525-2533.
20. Bichler J, Herrmann RG: **Analysis of the promotors of the single-copy genes for plastocyanin and subunit delta of the chloroplast ATP synthase from spinach**. *Eur J Biochem* 1990, **190**:415-426.
21. Pastuglia M, Roby D, Dumas C, Cock JM: **Rapid induction by wounding and bacterial infection of an S gene family receptor-like kinase in *Brassica oleracea***. *Plant Cell* 1997, **9**:1-13.
22. Kim JK, Cao J, Wu R: **Regulation and interaction of multiple protein factors with the proximal promoter regions of a rice high pI alpha-amylase gene**. *Mol Gen Genet* 1992, **232**:383-393.
23. Kuhlemeier C, Cuozzo M, Green PJ, Goyvaerts E, Ward K, Chua NH: **Localization and conditional redundancy of regulatory elements in rbcS-3A, a pea gene encoding the small subunit of ribulose-bisphosphate carboxylase**. *Proc Natl Acad Sci USA* 1988, **85**:4662-4666.
24. Pastuglia M, Roby D, Dumas C, Cock JM: **Rapid induction by wounding and bacterial infection of an S gene family receptor-like kinase in *Brassica oleracea***. *Plant Cell* 1997, **9**:1-13.
25. Kim SY, Wu R: **Multiple protein factors bind to a rice glutelin promoter region**. *Nucleic Acids Res* 1990, **18**:6845-6852.
26. Grandbastien MA, Berry-Lowe SL, Shirley BW, Meagher RB: **Two soybean ribulose-1,5-bisphosphate carboxylase small subunit genes share extensive homology even in distant flanking sequences**. *Plant Mol Biol* 1986, **7**:451-465.
27. Kwon HB, Park SC, Peng HP, Goodman HM, Dewdney J, Shih MC: **Identification of a light-responsive region of the nuclear gene encoding the B subunit of chloroplast glyceraldehyde 3-phosphate dehydrogenase from *Arabidopsis thaliana***. *Plant Physiol* 1994, **105**:357-367.
28. Itzhaki H, Woodson WR: **Characterization of an ethylene-responsive glutathione S-transferase gene cluster in carnation**. *Plant Mol Biol* 1993, **22**:43-58.
29. Diaz-De-Leon F, Klotz KL, Lagrimini M: **Nucleotide sequence of the tobacco (*Nicotiana tabacum*) anionic peroxidase gene**. *Plant Physiol* 1993, **101**:1117-1118.

(B) Positions of *cis*-acting elements for each Os*NIP* gene in rice


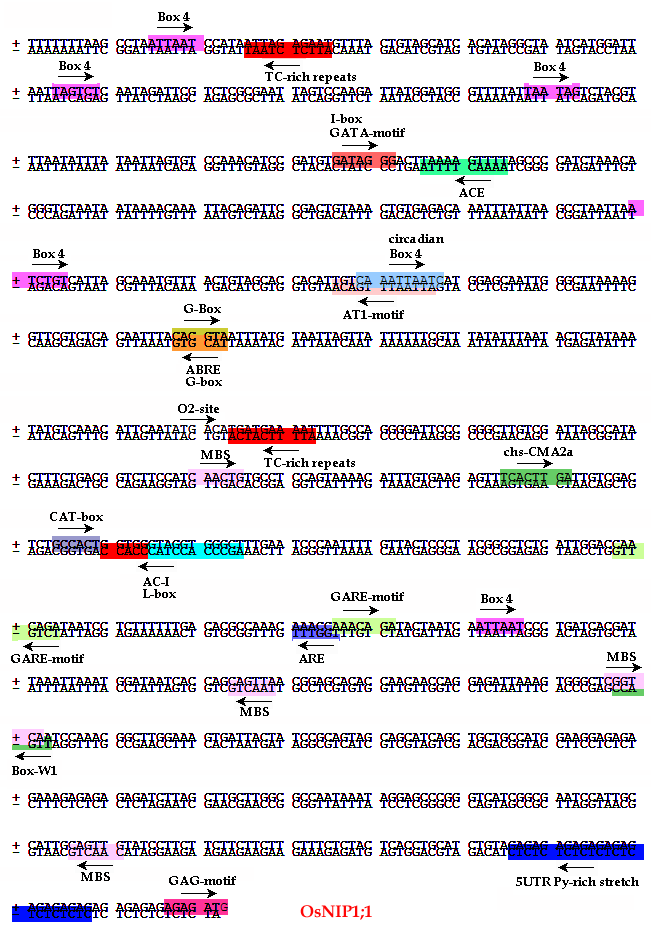


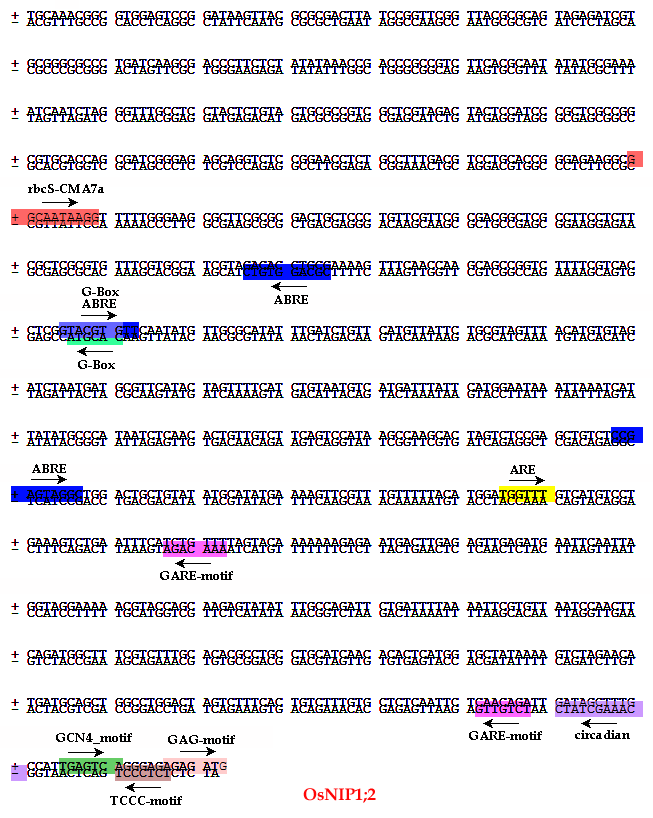


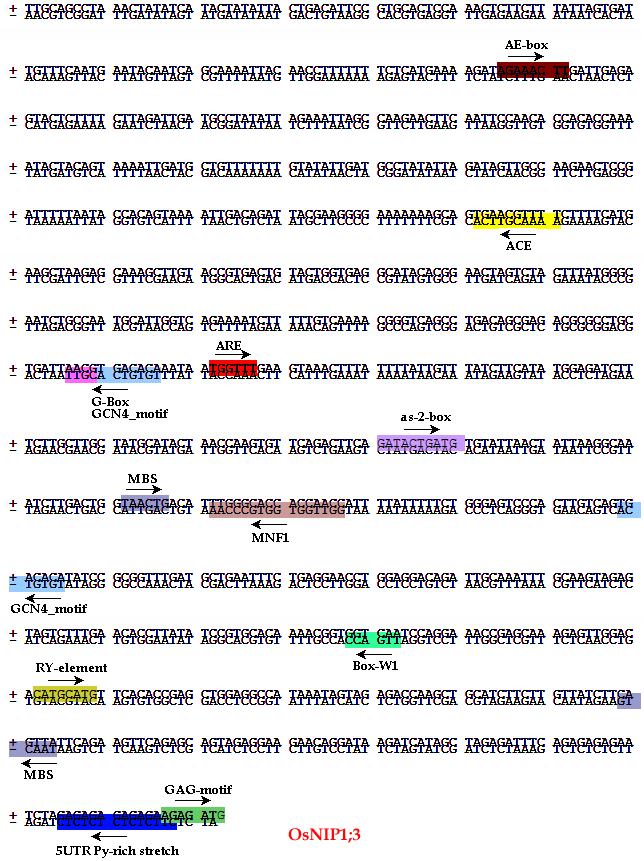


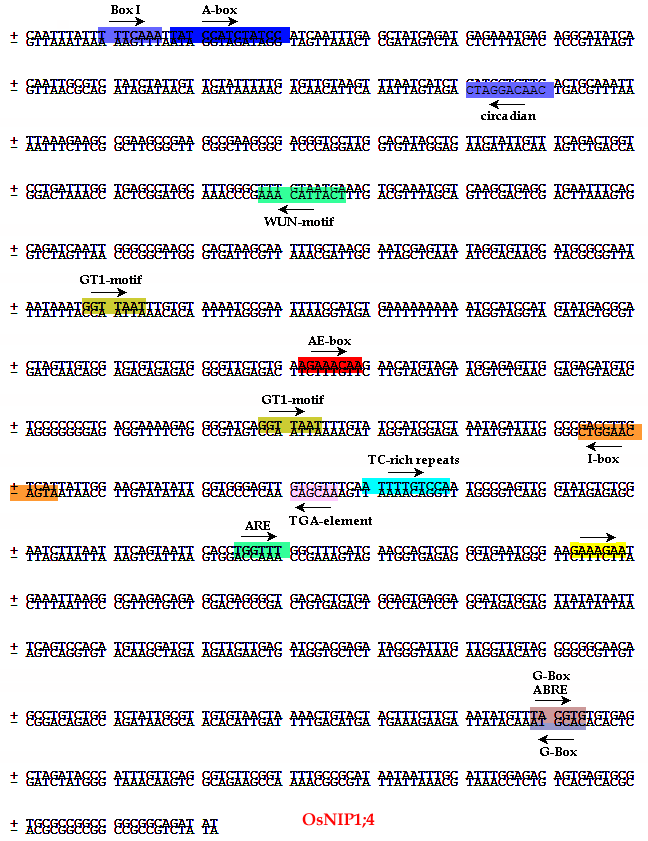


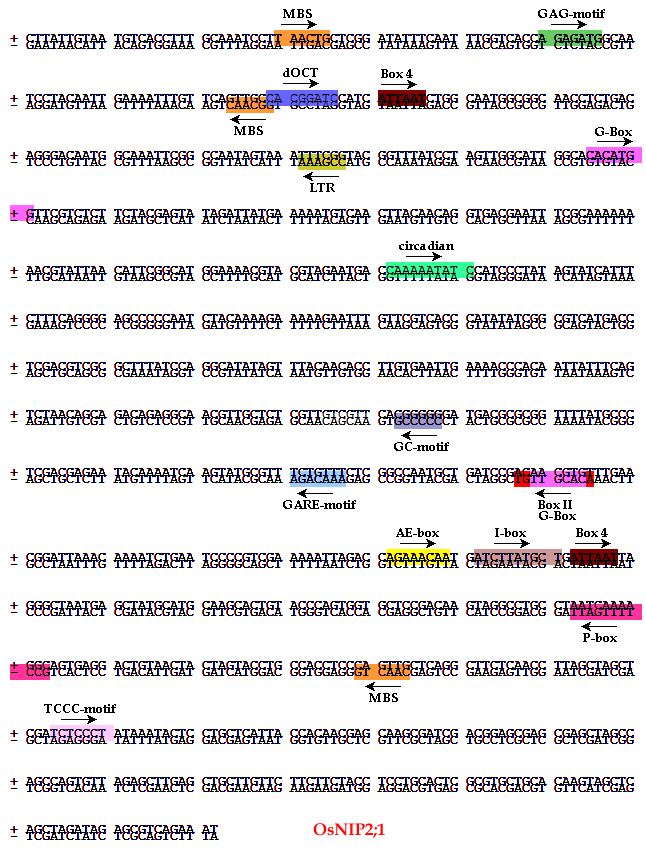


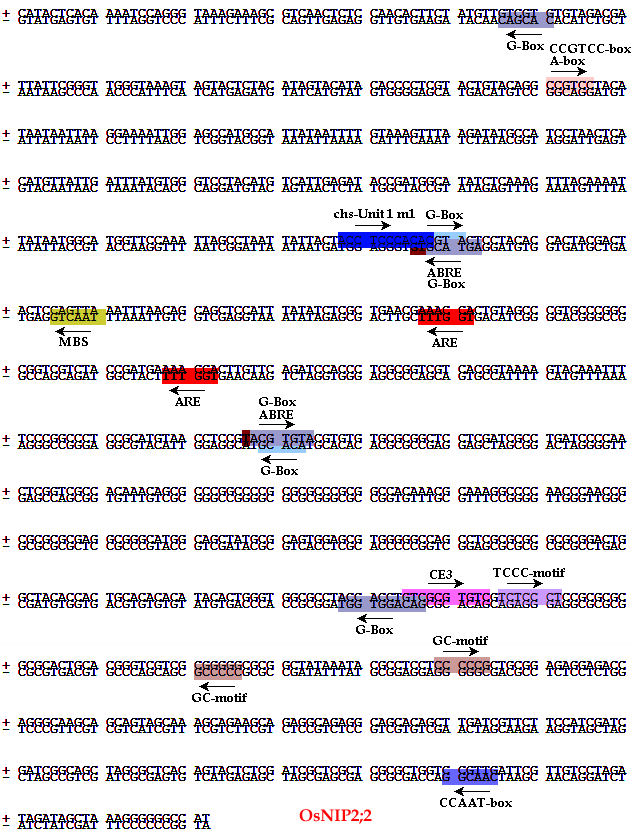


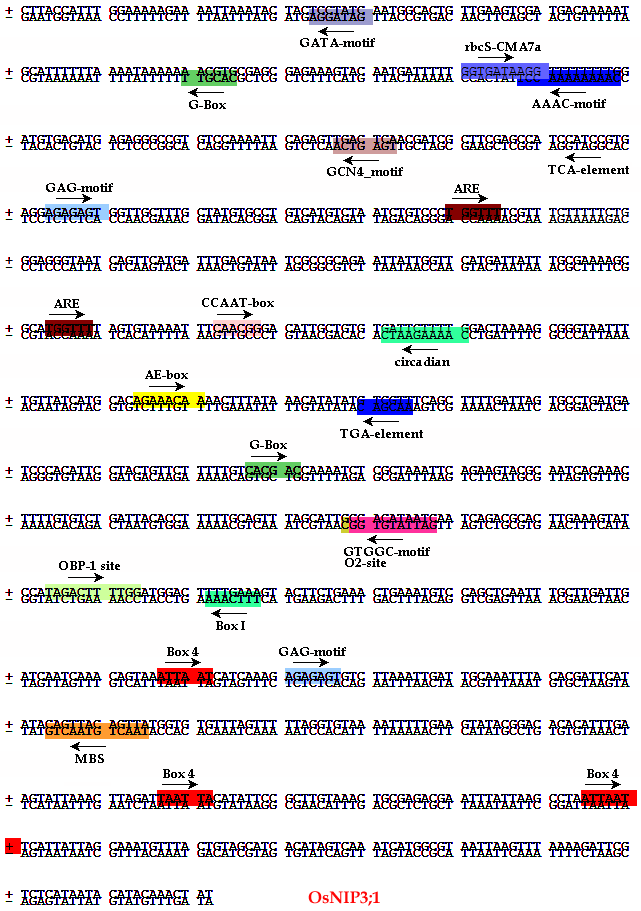


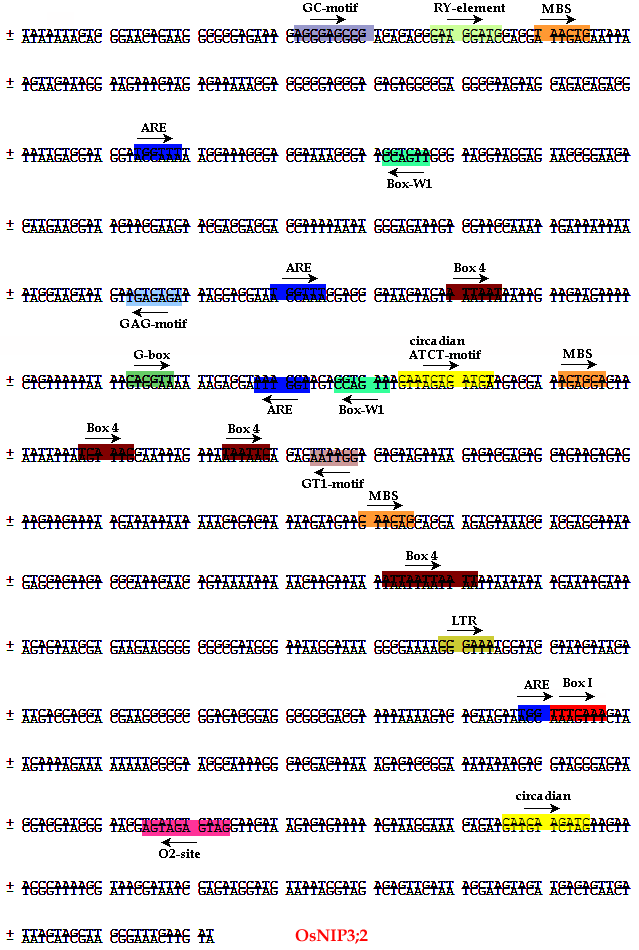


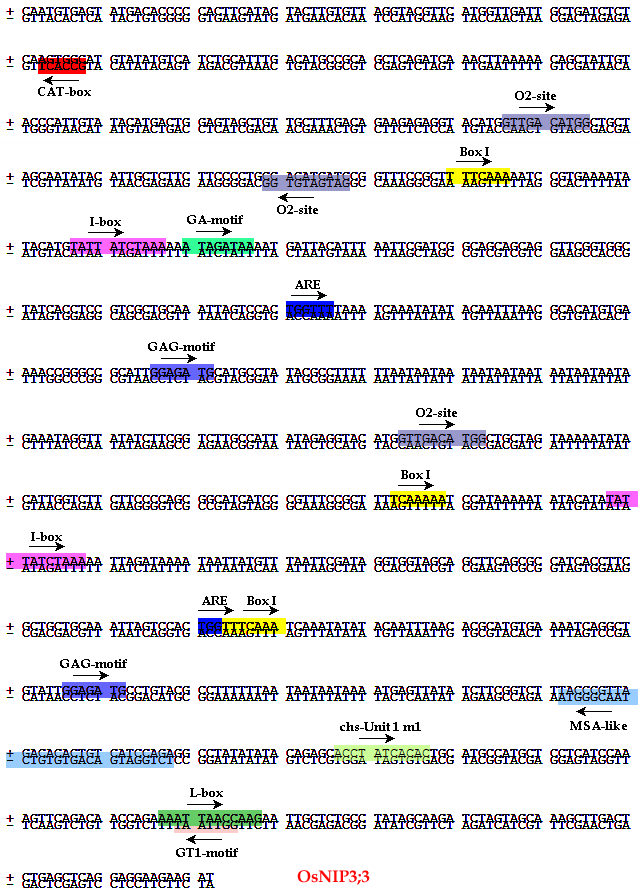


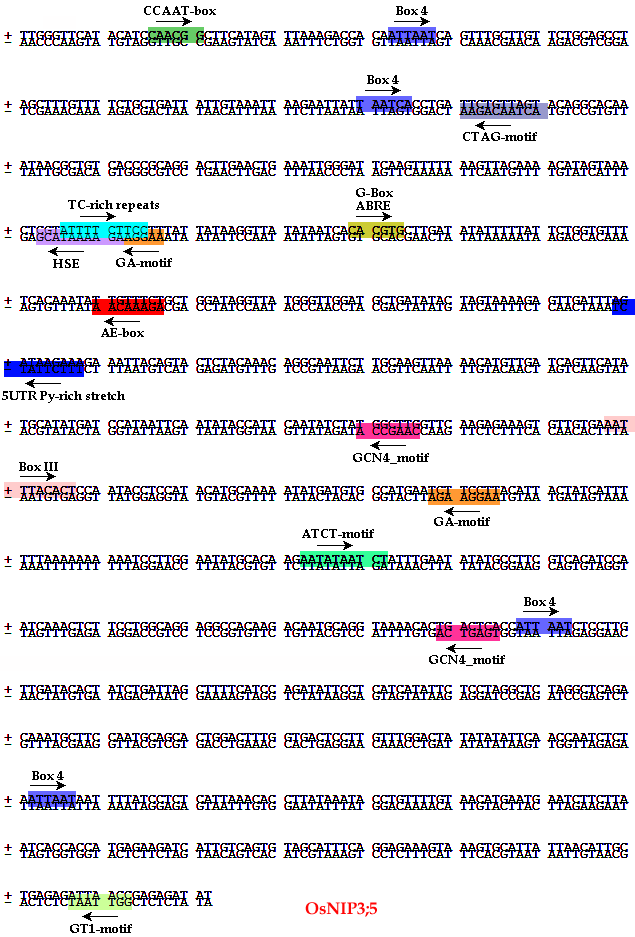


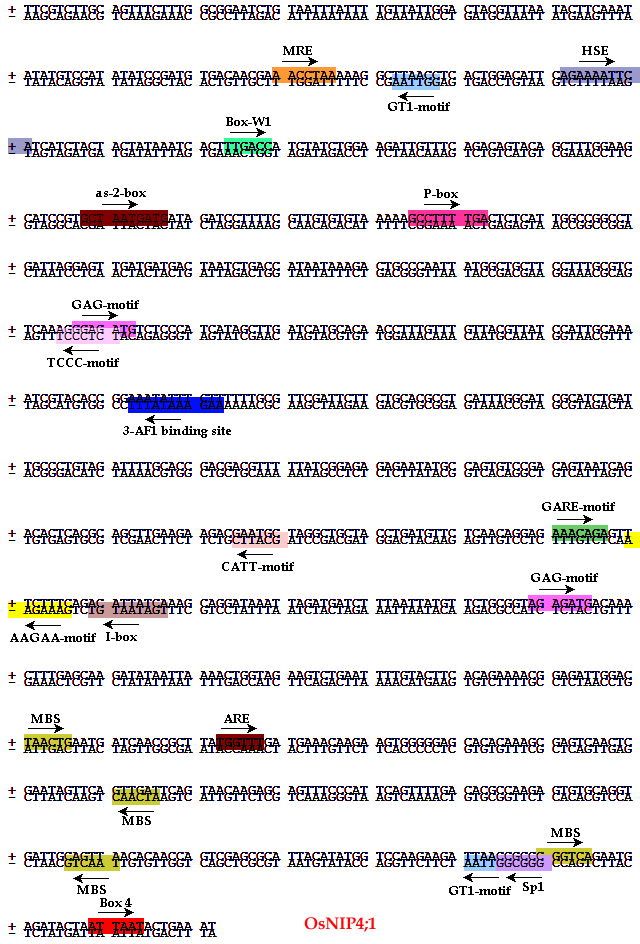

Supplement: Additional file 7 — Analysis of cis-acting elements in the 1000 bp sequence upstream of the translation initiation codon in plant NIP genes. [file 1471-2164-10-313-S7.doc]
